# Titanium Nanosurface with a Biomimetic Physical Microenvironment to Induce Endogenous Regeneration of the Periodontium

**DOI:** 10.1021/acsami.2c06679

**Published:** 2022-06-13

**Authors:** Masahiro Yamada, Tsuyoshi Kimura, Naoko Nakamura, Jun Watanabe, Nadia Kartikasari, Xindie He, Watcharaphol Tiskratok, Hayato Yoshioka, Hidenori Shinno, Hiroshi Egusa

**Affiliations:** † Division of Molecular and Regenerative Prosthodontics, Tohoku University Graduate School of Dentistry, Sendai, Miyagi 980-8575, Japan; ‡ Institute of Biomaterials and Bioengineering, Tokyo Medical and Dental University, Chiyoda-ku, Tokyo 101-0062, Japan; § Department of Bioscience and Engineering, College of Systems Engineering and Science, Shibaura Institute of Technology, Saitama, Saitama 337-8570, Japan; ∥ Laboratory for Future Interdisciplinary Research of Science and Technology, Tokyo Institute of Technology, Yokohama, Kanagawa 152-8550, Japan; ⊥ Center for Advanced Stem Cell and Regenerative Research, Tohoku University Graduate School of Dentistry, Sendai, Miyagi 980-8575, Japan

**Keywords:** biomimetics, dental implants, *in situ* tissue regeneration, nanotechnology, periodontium

## Abstract

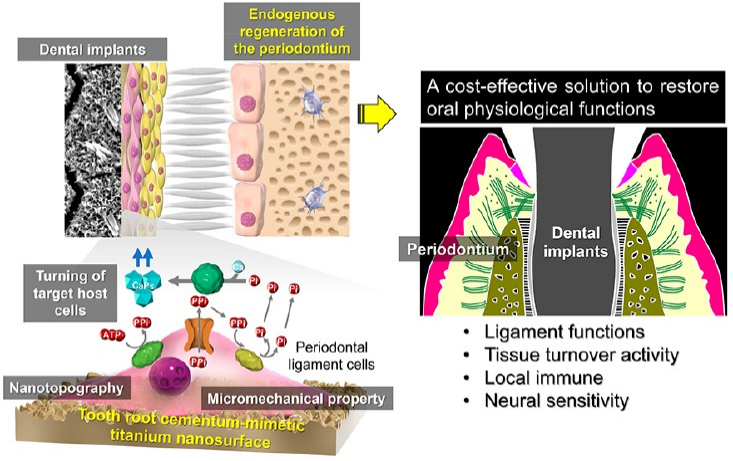

The periodontium supports the teeth by dentoalveolar fibrous joints that serve unique oral functions. Endogenous regeneration of the periodontium around artificial teeth (dental implants) provides a cost-effective solution for the extension of healthy life expectancy but remains a challenge in regenerative medicine. Biomimetics can create smart biomaterials that tune endogenous cells at a tissue–material interface. Here, we created a smart titanium nanosurface mimicking the surface nanotopography and micromechanical properties of the tooth root cementum (TRC), which is essential for the induction of dentoalveolar fibrous joints to regenerate the periodontium. After transplantation into the rat renal capsule, only the titanium artificial tooth with the TRC-mimetic nanosurface formed a complex dentoalveolar fibrous joint structure, with bone tissue, periodontal ligament (PDL), and TRC, in the decellularized jawbone matrix. TRC-mimetic titanium implants induce the formation of functional periodontium, even in a jawbone implantation model, which generally causes osseointegration (ankyloses). In human PDL cells, TRC analogousness in the surface mechanical microenvironment regulates matrix mineralization through bone sialoprotein expression and phosphorus metabolism, which are critical for cementogenesis. Therefore, the titanium nanosurfaces with nanotopographical and mechanical microenvironments mimicking the TRC surface induce dentoalveolar fibrous joints for periodontal regeneration by interfacial tuning of endogenous cells.

## Introduction

Tooth loss is among the top 100 causes of disability-adjusted life years.^1^ Osseointegrated dental implants are bone-anchored therapeutic titanium devices to replace missing teeth but they cannot regenerate periodontium. The periodontium is characterized by dentoalveolar fibrous joints that comprises mineralized tooth root cementum (TRC) and a periodontal ligament (PDL) intervening between the alveolar bone proper and TRC via Sharpey’s fibers. The PDLs serve as shock absorbers similar to other fibrous joints^2^ and support the tooth under masticatory loading. The PDLs also provide a niche for various cells, including immune cells, osteoblasts, fibroblasts, and progenitor/stem cells. These heterogeneous resident cells induce alveolar bone remodeling, adjusting the tooth position in response to externally applied forces or jawbone growth.^3^ In addition, the periodontium has the distinctive host defense system associated with PDLs.^4^ Dentoalveolar fibrous joints are one of fibrous joints with the unique biomechanical and osteoimmunological functions that are essential for oral functions. However, dentoalveolar fibrous joints never regenerate after tooth extraction.^5^ Severe mechanical and biological complications often occur in dental implant therapy because of a lack of periodontium.^6^

Various tissue engineering approaches using dental follicle cells or PDL stem cells have attempted periodontal tissue regeneration on dental implants.^7,8^ Dental follicle cells originating from the cranial neural crest-derived dental mesenchyme differentiate into cementoblasts, fibroblasts, and osteoblasts to form dentoalveolar fibrous joints.^9^ PDL stem cells existing as a subset in heterogeneous periodontal ligament cells (PDLCs) are similar in character to mesenchymal stem cells (MSCs)^10^ and are involved in periodontal tissue regeneration by differentiating into three types of periodontal tissue-forming cells.^11^ However, the uses of these biological resources for regeneration of the periodontium have technical, ethical, or economic issues.^12^ In addition, periodontal regeneration on dental implants requires spatially controlled differentiation of endogenous stem/progenitor cells, placing the proper cell type in the proper region, to form the distinguishing trilaminar ligament structure.^13^

Endogenous regeneration of damaged tissues or organs is less invasive and more cost-effective than tissue regeneration induced by exogenous cell transplantation.^14^ Endogenous regeneration requires manipulating endogenous resident cells or their niche.^15^ In situ tissue regeneration has attracted great attention as a biomaterial-based approach to induce endogenous regeneration by activating the body’s inherent regenerative capability without grafting exogenous cells.^16^ At the core of the concept is designing a biomaterial that recruits endogenous progenitor/stem cells to the proper local tissue and differentiates them into target tissue-forming cells.^16^ The principle is based on the fact that cells exert optimal function by sensing the surrounding microenvironment.^17^ Interestingly, the microenvironment to regulate stem cells includes not only ligand stimulations, such as growth factors, cytokines, and extracellular matrix (ECM), but also artificial physical cues, such as random/anisotropic nanotopography^18^ or micromechanical properties.^19^ Those artificial mechanical cues can affect the epigenetic processes of cellular differentiation as long as the cells are on the substrate.^20^ Those imply the possibility of in situ tissue regeneration by modifying surface physical properties of biomaterial.

Biomimetics is an approach to creating functionalized smart materials by mimicking the morphology and physicochemical properties of living tissues.^21^ Titanium dental implants never allow periodontal tissue regeneration in fresh extraction sockets, even where PDLs remain.^5^ In contrast, autologous tooth roots with an intact TRC and a persisting PDL can avoid ankylosis and reconstruct the trilaminar structure of dentoalveolar fibrous joints within extraction sockets or prepared bony cavities. In particular, the differentiation of resident PDL stem cells into cementoblasts is important for endogenous regeneration of the periodontium.^22^ The microenvironment on the tooth root including the TRC might control PDL stem cell differentiation into the proper cell type in the proper region to form the dentoalveolar fibrous joint structure in the periodontium.^23^ The strategy to control the differentiation of endogenous PDL stem cells leading to in situ regeneration of the periodontium around dental implants involves creating a pseudo microenvironment of the TRC on the dental implant surface. The alkali-etching treatment has a potential to create a titanium spiky nanosurface that tunes various types of cells such as macrophages^24,25^ and fibroblasts^26,27^ to exert functions suitable for the surrounding environment. Here, we introduce a novel, smart titanium nanosurface mimicking the nanotopography and micromechanical properties of the TRC surface using this simple titanium surface modification method. The present study demonstrated that the TRC-mimetic titanium surface promotes the differentiation of endogenous PDLCs into cementoblasts at the tissue–material interface and achieves in situ regeneration of the functional periodontium on dental implants as with autologous tooth transplantation, without using exogenous biological resources such as stem cells or growth factors.

## Results and Discussion

**Figure 1:**
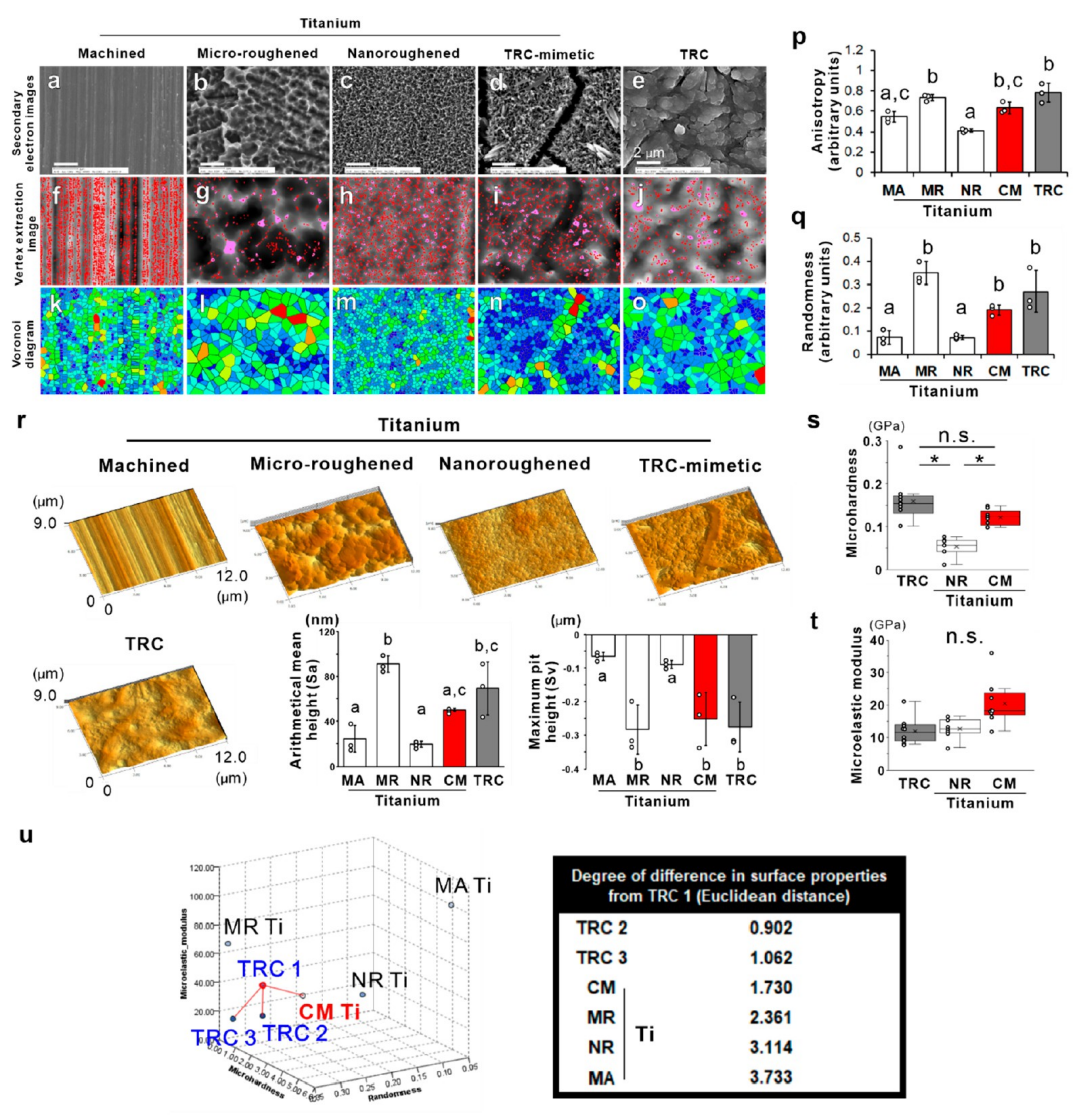
Topographic and micromechanical features of TRC and titanium surfaces. Secondary electron images under SEM and corresponding vertex extraction images and Voronoi diagrams of (a, f, and k) machined (MA), (b, g, and l) microroughened (MR), (c, h, and m) nanoroughened (NR), and (d, i, and n) TRC-mimetic (CM) titanium surfaces and (e, j, and o) TRC, respectively. (p) Anisotropy and (q) randomness in the vertex distribution on titanium and TRC surfaces were measured by analyzing vertex extraction images and Voronoi diagrams. (r) Bird’s-eye view of 3D reconstruction images (panels) and vertical roughness parameters (histograms) on titanium and TRC surfaces by 3D SEM. Three independent regions on one sample. Data are presented as the mean ± SD with dot plots. Different letters indicate statistically significant differences between them (*p* < 0.05; Tukey’s HSD test). (s) Microhardness and (t) microelastic modulus measured by nanoindentation on NR and CM titanium surfaces and TRC surfaces. Seven to ten independent spots on one surface. Data are presented as box and dot plots with a mean point. *Statistically significant differences among NR and CM titanium and TRC surfaces (*p* < 0.05; Tukey’s HSD test) (s). n.s. indicates no **s**tatistically significant differences between TRC and CM titanium surfaces (*p* > 0.05; Tukey’s HSD test) (s) or among TRC and NR and CM titanium surfaces (*p* > 0.05; Tukey’s HSD test) (t). (u) Multidimensional plots (left) of the horizontal pattern of vertex distribution, vertical roughness, and micromechanical properties of TRC1–TRC3 and titanium disks with an MA, MR, NR, or CM surface. The Euclidean distance (right) between TRC1 and others measured by *k* nearest neighbor analysis on multidimensional plots. TRC, tooth root cementum; SEM, scanning electron microscopy; HSD, honestly significant difference; SD, standard deviation.

Scanning electron microscopy (SEM) showed that typical surfaces of titanium dental implants include machined (Figure 1a), micro- (Figure 1b), or nanoroughened (Figure 1c) surfaces as linear turning grooves, irregular sharp ridges with micron pits, or numerous uniformly distributed nanospikes with nanoholes, respectively. In contrast to nanoroughened titanium surfaces produced by a similar alkali-etching treatment with sodium hydroxide (NaOH), the TRC-mimetic titanium surface (Figure 1d) showed numerous, dense, but nonuniformly distributed nanospikes with irregularly spread nanocrevices. The human TRC surface (Figure 1e) showed rounded, irregular undulations with cracks of different sizes. Horizontal spatial analysis of the vertex extraction image (Figure 1f–j) showed that the number of vertexes detected is much higher on the titanium surfaces, except for the microroughened titanium surfaces, compared to the TRC surface (Figure S1a). The Voronoi diagram^24^ and quadrat analysis (Figure 1k–o) showed that the geometric anisotropy and randomness on TRC-mimetic and microroughened titanium surfaces are not different from those on the TRC surface (Figure 1p, q). These results indicated that TRC-mimetic titanium surfaces have a horizontal pattern of vertex distribution fractal to the TRC surface. Microroughened and TRC-mimetic titanium surfaces also had similar representative vertical roughness parameters as TRC surfaces, as evaluated by 3D SEM analysis, in contrast with machined and nanoroughened titanium surfaces (Figure 1r and Figure S1b and c).

The machined titanium surface had incomparably higher microhardness and microelastic modulus determined by nanoindentation compared to TRC and nanoroughened titanium surfaces (Figure S1d, e). The microhardness and the microelastic modulus of the microroughened titanium surface were lower than those of the machined titanium surface, but not low enough to reach the levels of the other surfaces. In contrast, the TRC-mimetic titanium surface was not significantly different from the TRC in both microhardness and microelastic modulus (Figure 1s, t).

The *k*-nearest-neighbor (KNN) method quantified the analogousness in surface properties between each titanium surface and TRCs with regard to the horizontal pattern of vertex distribution, the vertical roughness, and the micromechanical properties as the Euclidean distance between dots in multidimensional plots. The analogousness to TRC of the TRC-mimetic titanium surface was close to that for other individual TRCs (Figure 1u) having similar topographical and micromechanical surface properties to each other (Figure S2a–c). These data prompted us to further explore the crystallographic features of the TRC-mimetic titanium surface and whether it regulates the stem cell differentiation as if it were a TRC surface.

**Figure 2:**
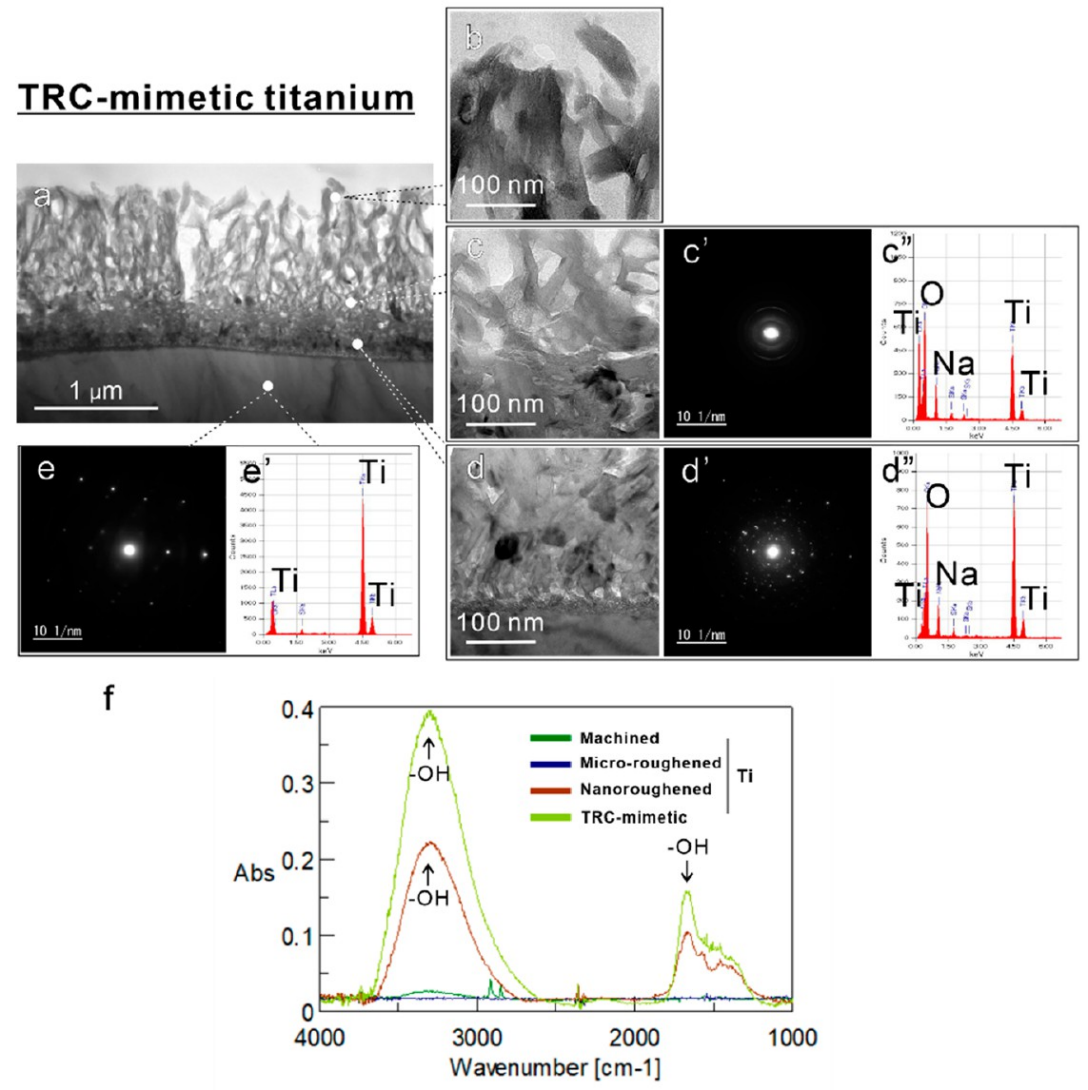
Topographic and micromechanical features of TRC and titanium surfaces. (a–d) Bright-field and (c′, d′, e) SAED images and (c″, d″, e′) EDX profile on the ultrathin titanium longitudinal section of TRC-mimetic titanium surfaces under TEM. Data were taken from each spot showing the corresponding typical features on (b, c–c″) superficial and (d–d″) transitional layers and (e, e′) titanium base. (f) Microreflection spectrum of infrared spectra on machined, microroughened, nanoroughened, and TRC-mimetic titanium surfaces. Note that the peaks for hydroxyl groups (−OH) were detected on both nanoroughened and TRC-mimetic titanium surfaces (black arrows). SAED, selected area electron diffraction; EDX, energy-dispersive X-ray spectroscopy; TRC, tooth root cementum; TEM, transmission electron microscopy.

Transmission electron microscopy (TEM) of ultrathin, longitudinal sections showed that the TRC-mimetic titanium surface consists of a spongy and shaggy superficial layer followed by a dense transitional layer to a titanium base (Figure 2a). The superficial layer was ∼1 μm thick, with some channels reaching the transitional layer, and had an amorphous-like crystalline structure that tended to show a preferred orientation toward the outermost layer and a lattice spacing value matching brookite-type titanium oxide, as shown by electron diffraction (ED) (Figure 2b, c, c′). The transitional layer showed a randomly oriented, relatively greater crystallized and denser structure of oxidized titanium (Figure 2d, d′) transition into a titanium base with an equally spaced and large crystal lattice (Figure 2e, e′). Energy-dispersive X-ray spectroscopy (EDX) detected sodium, oxide, and titanium atoms on the superficial layer (Figure 2c″, d″). These results indicated the coexistence of sodium titanate and brookite-type titanium oxide after alkali-etching treatment with NaOH as previously reported.^28^ The nanoroughened titanium surface (Figure S3a–d′) was sparse with a superficial shaggy and spongy structure and thin transitional layer but had the almost same crystallographic and atomic features as the TRC-mimetic titanium surface. Fourier transform infrared (FTIR) spectroscopy detected peaks for hydroxyl groups on alkali-etching-treated (nanoroughened or TRC-mimetic) titanium surfaces but not on machined and microroughened titanium surfaces (Figure 2f).

In a rodent model, in vivo reconstruction of dentoalveolar fibrous joints is observed in the mouse decellularized jawbone-tooth complex transplanted under the rat renal capsule,^23^ which is a potential niche harboring undifferentiated MSCs.^29^ Decellularized jawbone and teeth leave PDL matrices on the alveolar bone proper and TRC, respectively (Figure S4a). Undifferentiated MSCs differentiate into PDL stem cells and/or osteoblast, fibroblast, and cementoblast progenitors, and restore the periodontium along with recellularization of each periodontium component.^23^

**Figure 3:**
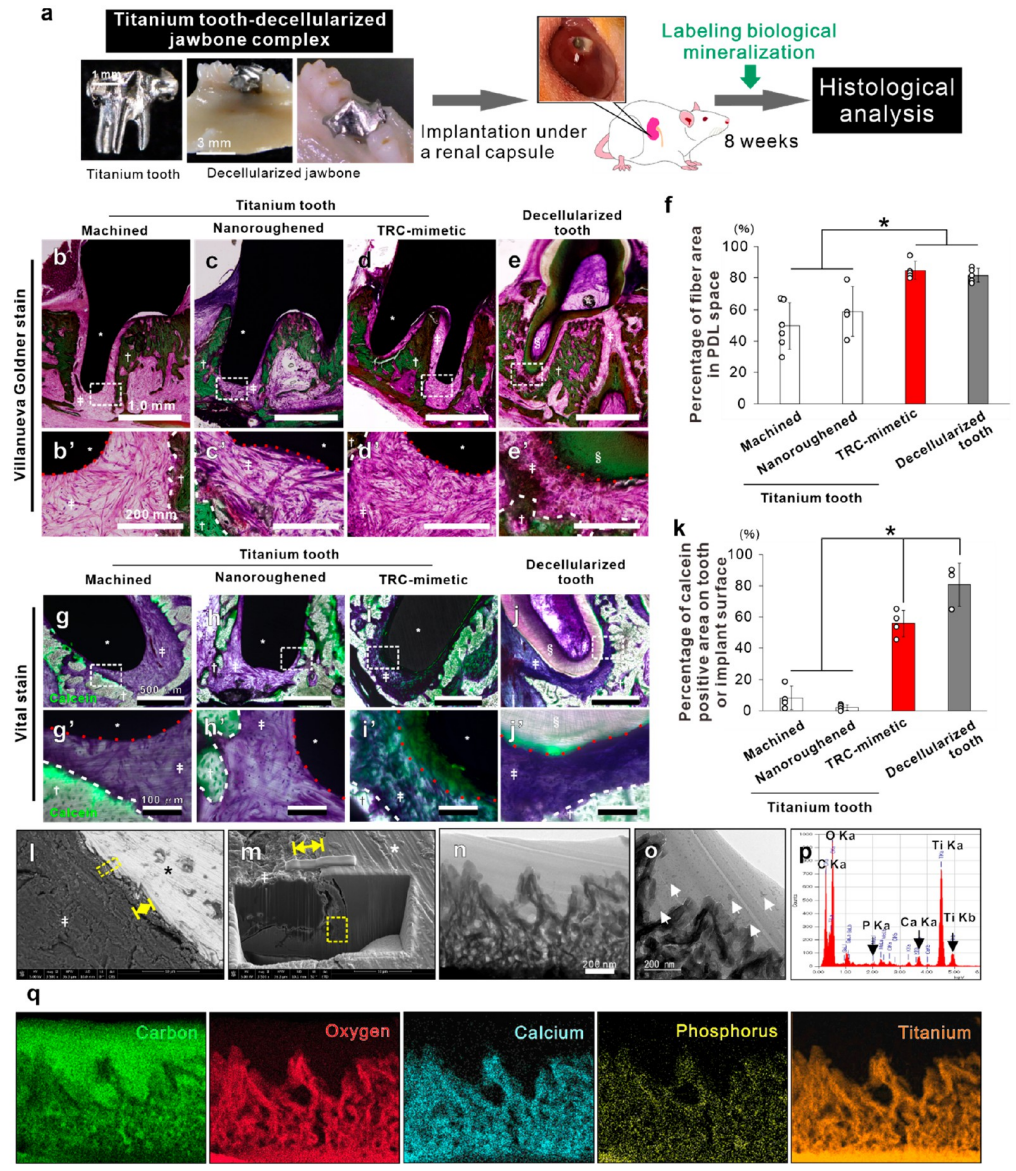
In vivo induction of dentoalveolar fibrous joints by TRC-mimetic titanium surfaces. (a) Schema showing the subrenal capsule transplantation model wherein a decellularized tooth or a titanium implant is inserted into the extraction socket of the decellularized jawbone and the complex is transplanted under the renal capsule of an 8-week-old male Wistar rats. (b–e′) Optical and (g–j′) fluorescent microscopic images on nondecalcified resi-embedded sections with Villanueva Goldner staining and Villanueva bone staining combined with calcein-based vital staining for the biological mineralization area, respectively, 8 weeks after subrenal capsule transplantation of the decellularized mandibular bone inserted with (e, e′, j, j′) a decellularized tooth or a tooth-shaped titanium implant with (b, b′, g, g′) machined, (c, c′, h, h′) nanoroughened, or (d, d′, i, i′) TRC-mimetic titanium surface. Images with the lowest magnification contain the entire tooth root or implant region (b–e and g–j), and highly magnified image sets show the interface between PDL-like-tissue and the TRC–titanium implant surface (b′–e′ and g′–j′). Green in fluorescent images (g–j′) indicates the biological mineralization area prelabeled by calcein-based vital staining. Percentages of fiber area in PDL space (f) and of the calcein-positive area on the tooth or implant surface (k) measured on optical and fluorescent microscopic images, respectively. Three to six independent animals on each tooth or titanium implant. Data are presented as mean ± SD with dot plots. *Statistically significant differences between the decellularized tooth and titanium implants, and machined, nanoroughened, or TRC-mimetic titanium surface (*p* < 0.05; Tukey’s HSD test). White dashed enclosures in the lowest magnified images (b–e and g–j) indicate the region for highly magnified images. White and red dashed lines indicate surfaces of the decellularized jawbone and the decellularized tooth root (TRC) or titanium implants, respectively. Bright-field images (n and o) and EDX profile (p) and mapping (q) under TEM on the ultrathin longitudinal section at the interface between the TRC-mimetic titanium implant and the PDL of nondecalcified resin-embedded samples 8 weeks after subrenal capsule transplantation. Yellow double arrow and dashed enclosures in the (l) backscattered and (m) secondary electron SEM images indicate the titanium implant–PDL interface and the points for cryo-FIB processing, respectively. Note (o) the punctate or linear matter (white arrows) suspected as residual PDL-like fibers after cryo-FIB processing on and outside the superficial layer and (p and q) detections of calcium and phosphorus in the entire region of the TRC-mimetic titanium surface at the interface with the biological tissue. *Titanium implant; ^†^decellularized jawbone, ^‡^PDL-like fibers; ^§^decellularized TRC. TRC, tooth root cementum; PDL, periodontal ligament; SD, standard deviation; HSD, honestly significant difference. TRC, tooth root cementum; EDX, energy-dispersive X-ray spectroscopy; TEM, transmission electron microscopy; PDL, periodontal ligament; SD, standard deviation; SEM, scanning electron microscopy; FIB, focused ion beam.

Custom-made tooth-shaped titanium implants with a machined, nanoroughened, or TRC-mimetic titanium surface (Figure S4b) or the mouse decellularized molar tooth was inserted into extraction sockets of the mouse decellularized mandibular jawbone and then this complex was transplanted under the rat’s renal capsule (Figure 3a). At week 8 post-transplantation, the TRC-mimetic titanium implants formed dense fibrous tissue connecting the implant surface and bone matrix (Figure 3d, d′) to a level equivalent to complete recovery around the decellularized tooth (Figure 3e, e′, f) in contrast with poor fiber formation on the other titanium implants (Figure 3b–c′). Those indicates that TRC-mimetic titanium restores PDL to its original architecture in coordination with decellularized PDLs and bone matrices by endogenous circulating stem cells, as seen around the decellularized tooth.

The TRC is cellular or acellular mineralized tissue covering the entire tooth root surface, and attach PDLs to the tooth root surface with extrinsic Sharpey’s fibers (ends of PDL fibers embedded into the TRC or alveolar bone proper).^30^ Cementogenesis is essential for the regeneration of dentoalveolar fibrous joints in the periodontium.^31^ TRC formation on titanium implants has never been achieved without transplantation of practically hard-to-use cells such as dental follicle cells^7^ and cementoblasts.^32^

At week 8 post-transplantation, a fluorescent signal of calcein as a vital staining dye for the biological mineralization area was detected in the decellularized bone matrix but little on machined and nanoroughened titanium surfaces (Figure 3g–h**′**). In contrast, as with the decellularized tooth root surface (Figure 3j, j′), TRC-mimetic titanium implants showed mineralizing signals over most of the surface (Figure 3i, i′, k). TEM analysis of an ultrathin longitudinal section at the interface between fibrous tissue and the TRC-mimetic titanium surface (Figure 3l–n) showed punctate or linear matter on and outside the superficial layer (Figure 3o). These structures should be the residual PDL-like tissue that was not blown off after cryo–focused ion beam (FIB) processing, because they were not found in the TEM section of the original surface (Figure 2b, c). EDX detected phosphorus (P) and calcium (Ca) atoms (Figure 3p, q), which did not originally exist (Figure 2c″, d″), from the entire titanium oxide layer. Therefore, the TRC-mimetic titanium surface induces dentoalveolar fibrous joints consisting of bone, PDL, and cementum on the surface by regulating the differentiation of the renal capsule-derived stem cells in coordination with decellularized jawbone and PDL matrices.

**Figure 4:**
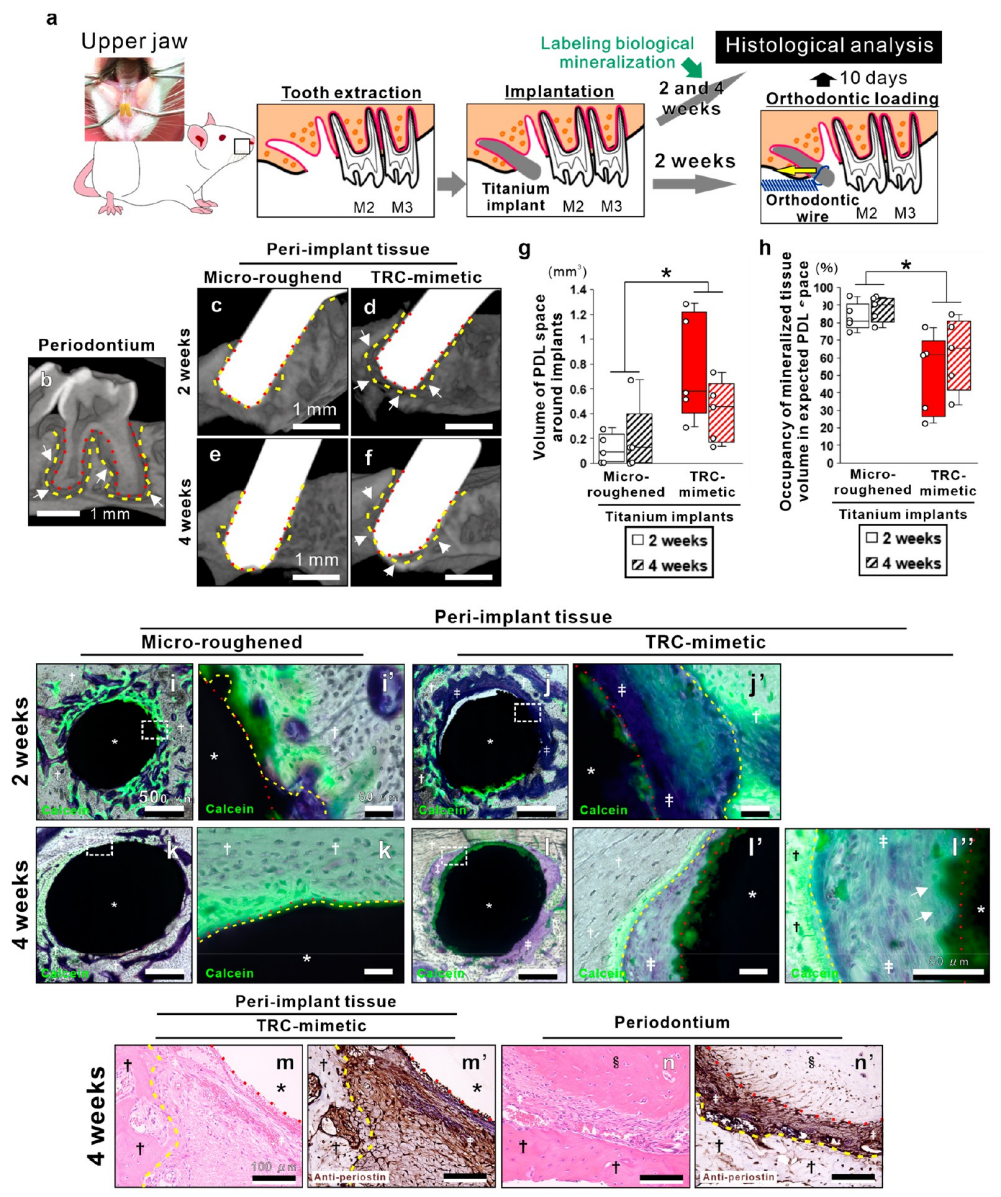
In situ regeneration of the periodontium with physiological function on TRC-mimetic titanium implants. (a) Schema showing the experimental design of the oral implant and orthodontic surgeries wherein titanium cylindrical mini-implants were placed into the mesial extraction socket with the residual PDL at the upper first molar (M1) of male Sprague–Dawley rats and underwent the orthodontic loading for functional analysis of peri-implant tissue. μCT images of (b) the periodontium around a natural molar tooth and peri-implant tissue around a titanium implant with (c and e) microroughened or (d and f) TRC-mimetic surface placed in the extraction sockets of the first molar tooth at 2 and 4 weeks. (g) PDL space volume around implants and (h) occupancy of mineralized tissue volume in expected PDL space by μCT analysis at 2 and 4 weeks postplacement. Five independent biological samples on each titanium implant. Data are presented as box plots with dot plots. *Statistically significant differences between titanium implants with microroughened and TRC-mimetic titanium surfaces (*p* < 0.05; Tukey’s HSD test). Fluorescent microscopic images of nondecalcified resin–embedded sections with Villanueva bone staining combined with calcein-based vital staining for the biological mineralization area at 2 and 4 weeks postplacement of titanium implants with (i, i′, k, k′) microroughened or (j, j′, l–l″) TRC-mimetic titanium surface. Images with the lowest magnification contain the entire region of the cross-sectioned peri-implant tissue (i, j, k, l), and highly magnified image sets show the implant surface–peri-implant tissue interface (i′, j′, k′, l′, l″). Green in fluorescent images indicate the biological mineralization area prelabeled by calcein-based vital staining. Optical microscopic images of paraffin-embedded decalcified sections stained with (m) hematoxylin-eosin and (m′) antiperiostin antibody on peri-implant tissue at the apical region of the TRC-mimetic titanium implant after 2 weeks’ healing. (n, n′) Histological images of the periodontal tissue of the molar at the apical region with the same staining shown as a reference. White dashed enclosures in the lowest magnified images (i, j, k, l) indicate the region for highly magnified images (i′, j′, k′, l′). Yellow and red dashed lines indicate surfaces of alveolar bone and TRC or titanium implants, respectively. *Titanium implant; ^†^alveolar bone; ^‡^PDL; ^§^tooth root. PDL, periodontal ligament; TRC, tooth root cementum; μCT, micro-computed tomography, HSD, honestly significant difference. Note the spaces around the TRC-mimetic titanium implant (d and f; white arrows) similar to the PDL space seen on a natural tooth (b; white arrows) in μCT images and the PDL fiber insertion into calcein-positive regions on the TRC-mimetic titanium implant surface (l″; white arrows).

Cylindrical titanium mini-implants with a microroughened or a TRC-mimetic titanium surface was placed into the mesial extraction sockets of a rat upper first molar (Figure 4a and Figure S5) of which some PDL remained on the alveolar bone proper (Figure S5) and, thus, where the implant surface could face the remaining PDLs or PDL stem cells. Even under such conditions, ossteointegration is accelerated, but neither PDL regeneration nor cementum formation is induced around microroughened titanium implants.^5^ At weeks 2 and 4 postplacement, micro-computed tomography (μCT) images and their 3D morphometry showed that microroughened titanium implants lose the PDL spaces separated by the alveolar bone proper and tooth root or implant surfaces (Figure 4c, e, g, h), which was found all on natural periodontium around the natural tooth root (Figure 4b). In contrast, the TRC-mimetic titanium implants kept the PDL space at weeks 2 and 4 postplacement (Figure 4d, f–h).

At weeks 2 and 4 postplacement, newly formed bone tissue connecting to the surrounding supportive bone was found, along with strong calcein signals within the expected PDL space and attached onto most surfaces of the microroughened titanium implants (Figure 4i, i′, k, k′). In contrast, at week 2 postplacement, the TRC-mimetic titanium implant surface allowed little bone deposition, with calcein signals and fibrous tissue intervening between the implant surface and the alveolar bone proper (Figure 4j, j′). At week 4 postplacement, the intensity of calcein signals overlapping onto most parts of the TRC-mimetic titanium implant surface increased (Figure 4l, l′), and the fibrous tissue containing cells was oriented toward the calcein-positive implant surface and the bundle bone with aligned, active osteoblast-like cells (Figure 4l″). In addition, the antibody of periostin, a representative matricellular molecule within PDLs,^33^ was intensively detected in the fibrous tissue between the TRC-mimetic titanium implant and the alveolar bone proper (Figure 4m, m′), as seen in PDLs of dentoalveolar fibrous joints in natural periodontium (Figure 4n, n′).

**Figure 5:**
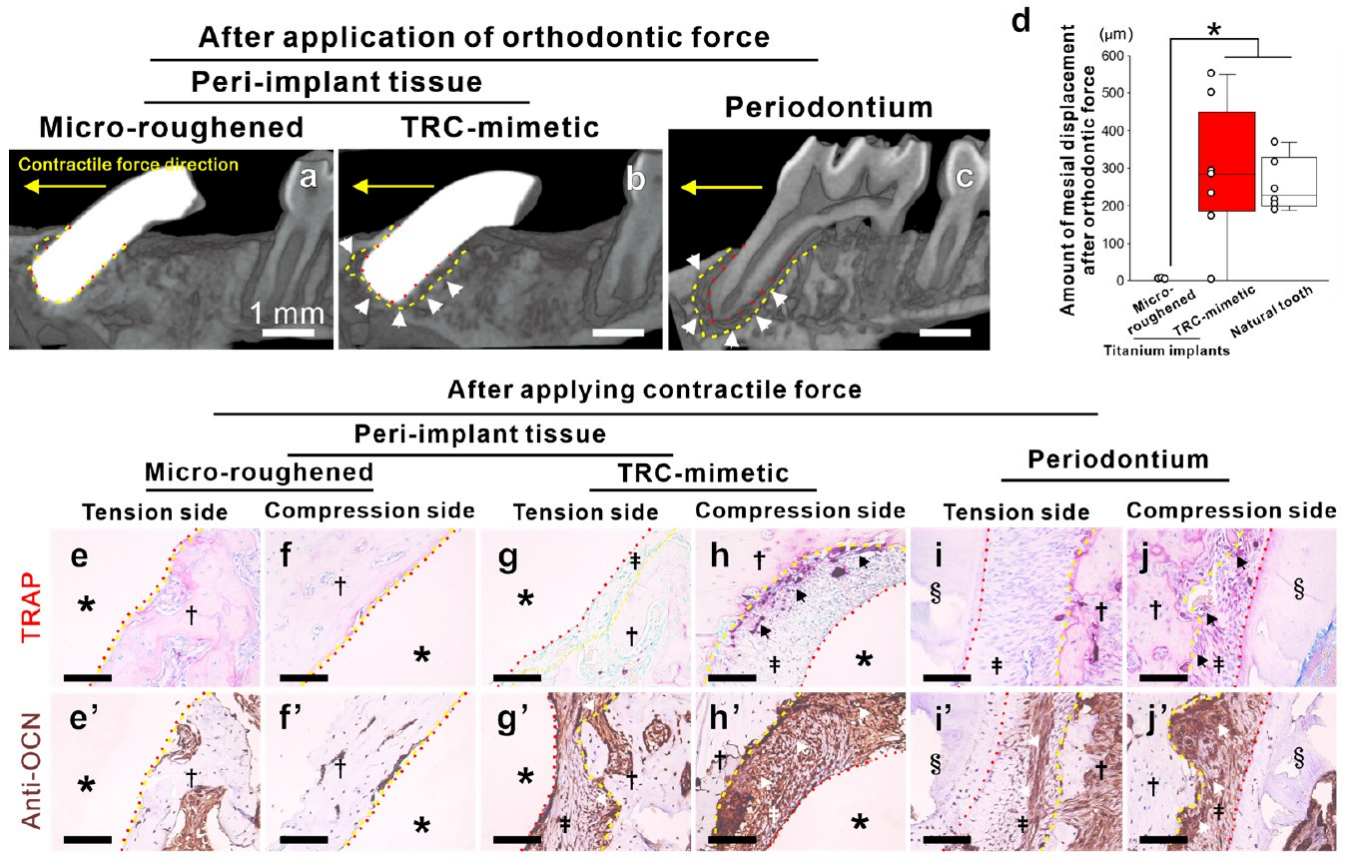
Physiological function in the periodontium regenerated on TRC-mimetic titanium implants. μCT images of peri-implant tissue around a titanium implant with (a) microroughened (osseointegrated) or (b) the TRC-mimetic titanium surface and (c) of the periodontium around a natural first molar tooth after application of a continuous contractile force in the distal-to-mesial (yellow arrow) direction for 10 days. Yellow and red dashed lines indicate surfaces of alveolar bone and tooth roots (TRC) or titanium implants, respectively. (d) Mesial displacement of the tooth and implants measured by μCT analysis of peri-implant tissue around titanium implants with microroughened or the TRC-mimetic titanium surface and of the periodontium around a natural first molar tooth after application of a continuous contractile force in the distal-to-mesial (yellow arrow) direction for 10 days. Three to five independent biological samples on each tooth or titanium implant. Data presented as box plots with dot plots. *Statistically significant differences between titanium implants with microroughened and TRC-mimetic titanium surfaces (*p* < 0.05; Tukey’s HSD test). Optical microscopic images of paraffin-embedded decalcified sections with (e–j) TRAP or (e′–j′) antiosteocalcin antibody staining on (e, e′, g, g′, i, i′) tensive distal and (f, f′, h, h′, j, j′) compressive mesial sides of (i, i′, j, j′) dentoalveolar fibrous joints of periodontium and of peri-implant tissue around the titanium implant with (e, e′, f, f′) microroughened or (g, g′, h, h′) TRC-mimetic surface, respectively. Note that the TRC-mimetic titanium implant keeps a space (b; white arrows) like the PDL space seen on a natural tooth (c) even after orthodontic force is applied. In addition, note that the detections of osteocalcin- (g′–j′; white arrows) and/or TRAP-positive cells (g–j; black arrows) are evident on the tension and compression sides of the peri-implant tissue around the TRC-mimetic implant after application of a contractile force, as seen in dentoalveolar fibrous joints of periodontium. TRC, tooth root cementum; μCT, micro-computed tomography; PDL, periodontal ligament; TRAP, tartrate-resistant acid phosphatase, HSD, honestly significant difference.

A distinctive function of dentoalveolar fibrous joints is to induce bone remodeling on the alveolar bone proper under an externally applied mechanical loading during orthodontic treatments, resulting in tooth displacement.^34^ At week 2 postplacement, the orthodontic force was applied on the implants in a distal-to-mesial direction (Figure 4a and Figure S5). Such directed orthodontic forces apply compressive and tensive forces via PDLs on the mesial and distal sides, respectively, of the alveolar bone proper around molar tooth roots.^35^ The mesial compression side generated osteoclasts with active osteoblasts, whereas the distal tension side predominantly generated active osteoblasts,^35^ resulting in mesial tooth displacement (Figure 5c). Even 10 days after orthodontic force application, the microroughened titanium implants kept the direct bone contact and were never displaced (Figure 5a). In contrast, the TRC-mimetic titanium implants kept PDL-like spaces with the alveolar bone proper (Figure 5b) and showed a mesial displacement similar to the natural tooth (Figure 5c), which was mesially displaced from 200 to 350 μm (Figure 5d). Osteoclasts and active osteoblasts, which are positive for tartrate-resistant acid phosphatase (TRAP) or osteocalcin, respectively, were rarely detected in the bone–implant interface of the microroughened titanium implants on either the tension or the compression side (Figure 5e–f′). In contrast, the TRC-mimetic titanium implants activated bone remodeling on both tension and compression sides of the alveolar bone proper (Figure 5g–h′) in response to orthodontic forces, as seen on natural periodontium (Figure 5i–j′). These results indicated that TRC-mimetic titanium implants promote regeneration of the periodontium with natural structure and function in contrast to promotion of bone attachment (osseointegration) on titanium implants.

**Figure 6:**
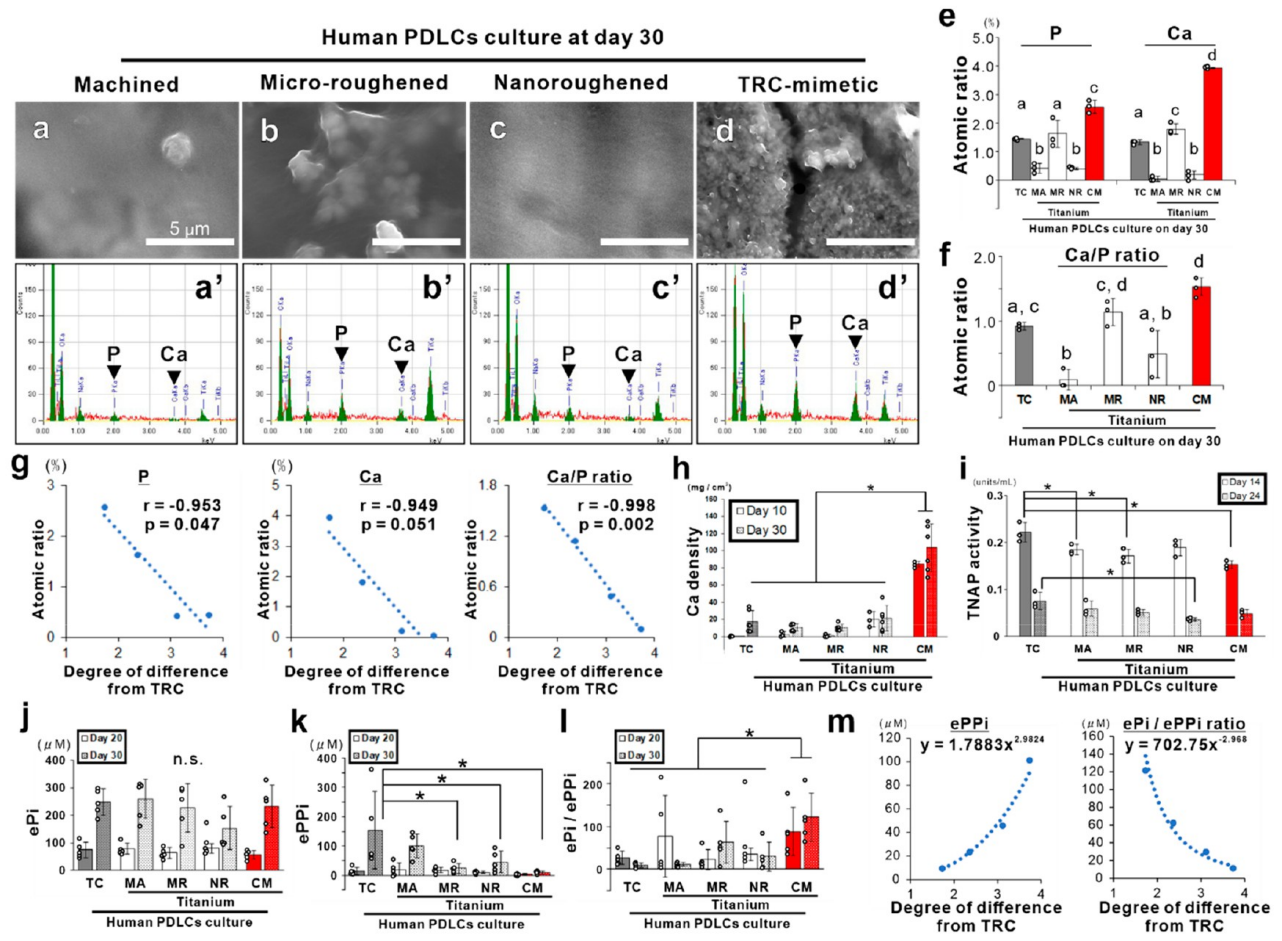
Regulation of matrix mineralization in hPDLCs for cementogenesis on titanium by mimicking the surface properties of TRC. (a–d) SEM images and (a′–d′) corresponding EDX profiles on hPDLC culture on titanium disks with (a and a′) machined (MA), (b and b′) microroughened (MR), (c and c′) nanoroughened (NR), or (d and d′) TRC-mimetic (CM) titanium surface in cementogenic induction medium for 30 days. (e) Atomic percentages of calcium (Ca) and phosphorus (P) and (f) the Ca/P ratio in hPDLC culture detected by EDX shown as the mean ± SD with dot plots. Data presented as the mean ± standard SD with dot plots. Three independent biological samples. Different letters indicate statistically significant differences between them (*p* < 0.05; Tukey’s HSD test). (g) Scatter plots with the Pearson correlation coefficient indicate a negative linear correlation between the mean value in the atomic percentage of Ca and P or in the Ca/P ratio and the degree of difference in surface properties from the tooth root shown in Figure 1u on each titanium implant surface. (h) Calcium density, (i) TNAP activity, (j) the amount of ePi, (k) the amount of ePPi, and (l) the ePi/ePPi ratio in hPDLC culture on a control polystyrene tissue culture (TC) plate or titanium disks with an MA, MR, NR, or CM surface, respectively, at days 10, 14, 20, 24, and/or 30 shown as the mean ± SD with dot plots. *Statistically significant differences between cultures on a titanium CM surface versus other implant surfaces (h) (*p* < 0.05; Tukey’s HSD test) and on a TC plate versus a titanium MA, MR, NR, or CM surface within or regardless of each time point (i–l) (*p* < 0.05; Dunnett’s test). n.s. indicates no statistically significant differences in ePi (j) among groups on any time points (*p* > 0.05; Tukey’s HSD test). Three to five independent biological samples. (m) Scatter plots indicate an exponential correlation between the mean value in the amount of ePPi or the ePi/ePPi ratio and the degree of difference in surface properties from the tooth root shown in Figure 1u on each titanium implant surface. hPDLCs, human periodontal ligament tissue-derived cells; PDL, periodontal ligament; TRC, tooth root cementum; SD, standard deviation; SEM, scanning electron microscopy; EDX, energy-dispersive X-ray spectroscopy; HSD, honestly significant difference; TNAP, tissue-nonspecific alkaline phosphatase; ePi, extracellular inorganic phosphate; ePPi, extracellular inorganic pyrophosphate.

Human PDL tissue-derived cells (hPDLCs) with matrix mineralization capability^36^ were cultured on machined, microroughened, nanoroughened, or TRC-mimetic titanium surfaces or tissue culture–treated polystyrene plates in osteogenic medium, which made hPDLCs express both osteoblastic and cementoblastic markers.^37^ At day 30, TRC-like pebbly nanosize spherical structures (Figure 6d) and the highest peaks for both P and Ca atoms (Figure 6d′, e) were detected only on the ECM on TRC-mimetic titanium surfaces in contrast with the featureless and poorly mineralized ECM on other titanium surfaces (Figure 6a–c′, e). The Ca/P ratio in hPDLC culture on TRC-mimetic titanium surfaces was ∼1.5 (Figure 6f), which was within the 1.3–1.65 range of human TRC.^38^ A strong negative linear correlation between the degree of difference in surface properties between each titanium surface and TRC (referenced in Figure 1u) and the atomic percentage of P and Ca atoms or the Ca/P ratio in the ECM of the hPDLC culture (Figure 6g) was also found. At days 10 and 30, the Ca density in hPDLC culture confirmed the higher matrix mineralization in the culture on the TRC-mimetic titanium surfaces than on the other surfaces (Figure 6h).

Although most tissue/gene markers are common between cementogenesis and osteogenesis,^39^ cementogenesis, particularly acellular cementum formation, is characterized by a specific matrix mineralization process based on directed mineralization of collagen fringe fibers at the tooth root surface.^40^ This process is largely dependent on physicochemical mineralization, which is exceptionally sensitive to local levels of extracellular inorganic phosphate (ePi) and inorganic pyrophosphate (ePPi).^41^ A high ePi/ePPi ratio promotes TRC defect healing.^42^ Local levels of ePi and ePPi are modulated by progressive ankylosis protein (ANK)^43^ and ectonucleotide pyrophosphatase/phosphodiesterase 1 (ENPP1).^44^ These transmembrane protein and glycoprotein are involved in the negative regulation of tissue mineralization^44^ and prevent hypercementogenesis of the acellular TRC by modulating local ePPi levels.^41^ In hPDLC culture, the activity of tissue-nonspecific alkaline phosphatase (TNAP), a key enzyme that hydrolyzes pyrophosphate into inorganic phosphate for matrix mineralization,^45^ was consistently almost the same or slightly lower on TRC-mimetic titanium surfaces compared to other implant surfaces (Figure 6i). In addition, the amount of ePi released from hPDLC culture was not different between any substrates day 20 or 30 (Figure 6j). However, the amount of ePPi released from hPDLC culture was consistently lower on TRC-mimetic titanium surfaces at days 20 and 30 (Figure 6k), and the ePi/ePPi ratio was consistently higher in hPDLC culture on TRC-mimetic titanium surfaces compared to other implant surfaces (Figure 6l). Both the amount of ePPi and the ePi/ePPi ratio were exponentially correlated with the degree of difference in surface properties between each titanium surface and the TRC (Figure 6m).

**Figure 7:**
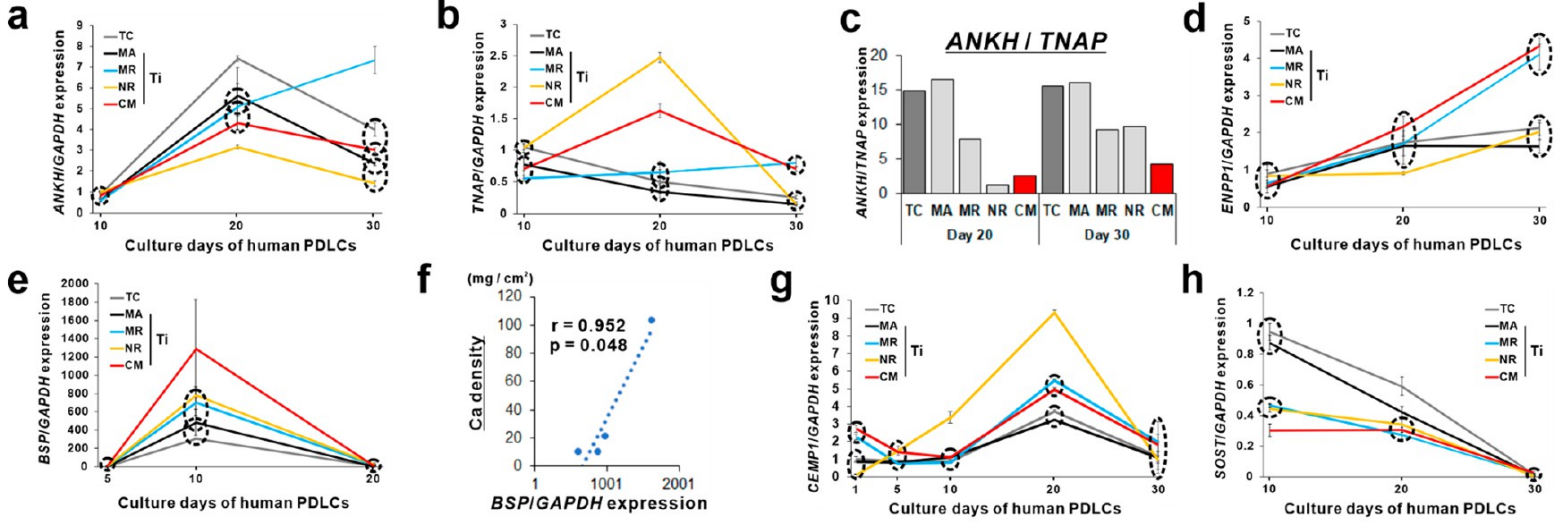
Regulation of hPDL cell differentiation toward periodontal tissue regeneration on titanium by mimicking the surface properties of TRC. Expression profiles of (a) *ANKH*, (b) *TNAP*, (d) *ENPP1*, (e) *BSP*, (g) *CEMP1*, and (h) *SOST* relative to *GAPDH* expression determined by RT-PCR in hPDLC culture on a control polystyrene tissue culture (TC) plate (gray) or titanium disks with an machined (MA; black), microroughened (MR; light blue), nanoroughened (NR; yellow), or TRC-mimetic (CM; red) surface at days 1, 5, 10, 20, and/or 30. Data are presented as line graphs with the mean ± SD. Three independent biological samples. Dashed circles indicate no statistically significant differences between surfaces within the same circle on each time point (*p* < 0.05; Tukey’s HSD test). (c) Ratio of the mean values of *ANKH* and *TNAP* expression on each surface at days 20 and 30 indicates consistent progression in ePPi consumption in hPDLC culture on a titanium CM surface. (f) Scatter plot with the Pearson correlation coefficient indicates a positive linear correlation between the mean values in the calcium density at day 30 in Figure 5h and (e) in *BSP* expression at day 10 in hPDLC culture. hPDLCs, human periodontal ligament tissue-derived cells; PDL, periodontal ligament; TRC, tooth root cementum; SD, standard deviation; HSD, honestly significant difference; *ANKH*, ankylosis protein homologue; *TNAP*, tissue-nonspecific alkaline phosphatase; *ENPP1*, ectonucleotide pyrophosphatase/phosphodiesterase 1; *BSP*, bone sialoprotein; *CEMP1*, cementoblastoma-derived protein 1; *SOST*, sclerostin; *GAPDH*, glyceraldehyde 3-phosphate dehydrogenase; RT-PCR, reverse transcription polymerase chain reaction.

In addition, phosphate metabolism-related genes in hPDLC culture showed specific expression profiles on TRC-mimetic titanium surfaces compared to other implant surfaces. Ankylosis protein homologue (*ANKH*) and *TNAP* expressions were coordinated on TRC-mimetic titanium surfaces, where both genes were upregulated at day 20 and were significantly upregulated even at day 30 (Figure 7a, b). The *ANKH*/*TNAP* expression ratio was always low on TRC-mimetic titanium surfaces compared to other implant surfaces, particularly at day 30 (Figure 7c). In addition, *ENPP1* was gradually upregulated in hPDLC culture on TRC-mimetic titanium surfaces in contrast with consistently low expression on other implant surfaces, except for microroughened titanium surfaces (Figure 7d). ENPP1 may play a specific role in mineralized tissue development, in addition to its catalytic activity.^46^ During tooth development, ANK is highly expressed in dental follicle cells and their differentiated cells,^47^ whereas ENPP1is highly expressed in the acellular TRC region during tooth root completion.^48^ Interestingly, ENPP1 is involved in osteoclastic activity responsible for orthodontic tooth movement.^49^ Therefore, the closer the surface properties, including topography and micromechanical properties, are to those of the TRC, the more the titanium surface promotes matrix mineralization by hPDLCs via control of eP/ePPi metabolism, which is important in physiological and functional TRC formation.

**Figure 8:**
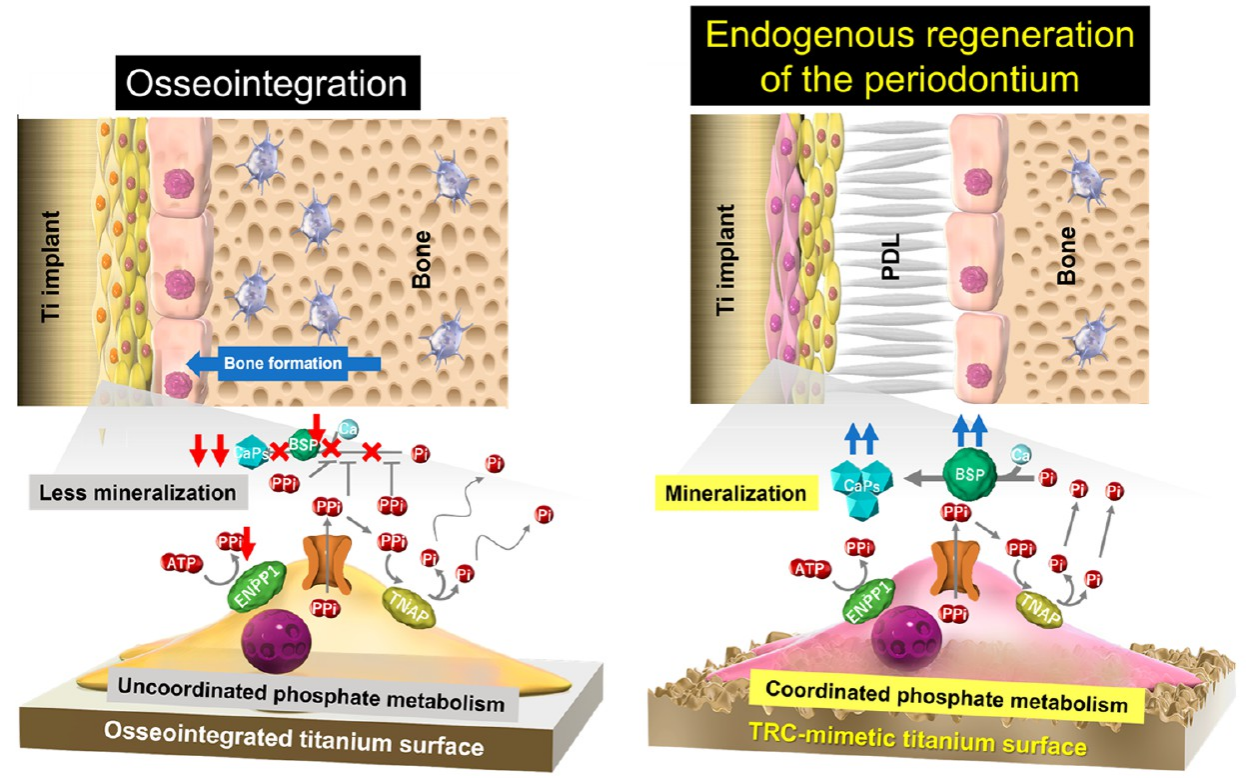
PDLC-mediated cementogenesis as the mechanism underlying the endogenous regeneration of periodontium on the TRC-mimetic titanium surface. A titanium implant surface with topographical and micromechanical properties mimicking those on the TRC surface (right panel) allows PDLCs to differentiate into cementoblast-like cells, promoting two key factors for matrix mineralization in cementogenesis, BSP expression and coordinated phosphate metabolism. Therefore, periodontal regeneration is established. In contrast, PDLCs on other titanium implant surfaces show disturbed BSP expression and phosphate metabolism, resulting in matrix mineralization failure (left panel). Simultaneously, osteoblasts on surrounding bone walls forward bone formation on residual PDLs as a scaffold, promoting osseointegration.

Bone sialoprotein (BSP) presents in mammalian hard tissues originating from the neural crest, such as the jawbone, some bones of the calvaria, the alveolar bone, and the TRC,^50^ from the early developmental stages.^51^ BSP is part of the small integrin-binding ligand, N-linked glycoprotein (SIBLING protein) family, such as osteopontin and dentin matrix protein 1, and functions as a nucleation center of hydroxyapatite (HAp).^52^ BSP is an essential component in early matrix mineralization for both osteogenesis and cementogenesis and is particularly fatal for acellular TRC and Sharpey’s fiber formation through the simultaneous process of matrix formation and mineralization.^51^ BSP expression in cementogenesis is independent of ANK and ENPP1 expressions.^43^ In hPDLC culture, *BSP* expression was transiently upregulated at day 10 on all substrates, but it was significantly higher on TRC-mimetic titanium surfaces compared to other implant surfaces (Figure 7e). The extent of *BSP* expression was correlated with the calcium density in hPDLC culture on titanium (Figure 7f). In addition, the TRC-mimetic titanium surfaces consistently up- or downregulated cementoblastoma-derived protein 1 (*CEMP1*) or sclerostin (*SOST*) in hPDLC culture, respectively, compared to polystyrene or machined titanium surfaces (Figure 7g, h). *CEMP1* is a specific gene marker for cementoblasts and a subpopulation of PDLCs,^53^ whereas *SOST* is a negative regulator for TRC and alveolar bone regeneration.^54^ Taken together with the results of in vivo and in vitro studies, TRC-mimetic titanium surfaces produced by a simple alkali-etching treatment for titanium, achieved in situ regeneration of the functional periodontium on the surface through the endogenous PDLC differentiation into cementoblasts with activated BSP expression and coordinated phosphate metabolism (Figure 8).

The alkali-etching treatment created the characteristic nanotopography depending on the concentration of NaOH or the boiling temperature^26,27^ regardless of the grade and shape of the base titanium (Figure 1 and Figures S4b and S5). The nanoroughened and TRC-mimetic titanium surfaces are made by alkali-etching treatment on machined titanium surfaces and have the same crystallographic and chemical features (Figure 2 and Figure S3). The difference between the two is only the analogousness to the TRC with respect to both surface nanotopography and micromechanical properties (Figure 1u). Interestingly, the analogousness to the TRC with respect to both surface nanotopography and micromechanical properties was correlated with the matrix mineralization and the phosphorus metabolism in the hPDLC culture on titanium surfaces (Figure 6g, m). Therefore, the closer surface properties, might be to the topographical and mechanical microenvironment of local tissue, the more likely the biomaterial induces in situ tissue regeneration by regulating the endogenous cellular differentiation.

It is still unclear which nanotopography or micromechanical properties are the primary factors affecting the biological capability of TRC-mimetic titanium nanosurfaces. The surface topography of the MR titanium surface was similar to that of TRC. However, the micromechanical properties markedly different between them (Figures 1 and S1). The MR titanium surface tended to upregulate *ANKH* and *ENPP1* expression involved in ePPi release in the hPDLC culture (Figure 7a and **d**). In contrast, the NR titanium surface was comparable with the TRC surface only in terms of the micromechanical properties (Figure 1) and showed favorable gene expression profiles for cementoblast differentiation of hPDLCs (Figure 7**a, b**, and **g**). However, all titanium surfaces, except for the TRC-mimetic surface, failed endogenous regeneration of the periodontium *in vivo* (Figures 3 and 4). Both nanotopography and micromechanical properties mimicking the TRC might be necessary to provide sufficient conditions for endogenous regeneration of the periodontium around titanium implants. Investigating the effects of each titanium surface property on the differentiation of hPDLCs would be of interest for future research.

Approximately 1 μm thickness of the superficial nanolayer of the TRC-mimetic titanium nanosurface (Figure 2a) was markedly thinner than several dozens to hundreds of micrometers of TRC thickness,^55^ although their micromechanical properties were the same (Figure 1**s and t**). Tooth roots sometimes suffer from cemental tears caused by excessive occlusal loading, leading to the breakdown of the periodontium.^56^ Thicker coatings are more prone to delamination than thinner ones.^57^ Similar to the microhardness and microelastic modulus (Figure 1**s and t**), our previous report showed that the interlaminar shear strength is higher on the TRC-mimetic titanium nanosurface than on the NR titanium surface.^26^ Although sufficient verification is required in the future, the structural and micromechanical properties of the TRC-mimetic titanium surface may be favorable as an artificial tooth root surface. However, the micromechanical properties of the TRC-mimetic titanium surface might be lower than that of jawbone tissue,^58^ potentially causing detachment of the surface nanostructure by friction with bone tissue, unlike machined or MR titanium surfaces. Furthermore, like natural tooth roots,^59^ TRC-mimetic titanium implants cannot tune osteoblastic lineage cells toward endogenous regeneration of the periodontium. For these biomechanical and biological reasons, endogenous regeneration of the periodontium around the TRC-mimetic titanium implants would require complete coverage of the implant surface with the PDL tissue. TRC-mimetic titanium implants might require exogenous cell transplantation for the periodontium regeneration in edentulous jawbones without the PDL tissue.

We found that, interestingly, PDL stem cells, one of the multipotent stem cells, sense the nanotopographical and micromechanical cues of the surrounding microenvironment and determine their own differentiation into a corresponding cell type. More importantly, this study is the first to report that titanium artificially mimics the physical microenvironment of biological cementum tissue with a simple alkali-etching treatment and can control the differentiation of endogenous stem cells to achieve endogenous regeneration of the functional periodontium. The proof-of-concept of endogenous tissue regeneration mediated by the biomimetic physical microenvironment of biomaterials paves the way toward a novel strategy for the development of smart biomaterials for tissue regeneration and interfacial tissue engineering.

## Conclusion

The titanium nanosurface with topographical and mechanical microenvironments mimicking a tooth root surface induces dentoalveolar fibrous joints to regenerate the periodontium by interfacial tuning of endogenous periodontal ligament cells. The titanium nanosurface regulates periodontal ligament cells to express the characteristics of cementoblasts and the coordinated phosphate metabolism required for matrix mineralization of the tooth root surface. Other types of titanium surfaces do not induce endogenous regeneration of the periodontium by disturbing cementoblast differentiation and/or phosphate metabolism in the periodontal ligament cells. Endogenous regeneration of the periodontium on titanium dental implants may require the analogousness of tooth root cementum in terms of both nanosurface topography and micromechanical properties. Although further investigations such as biomechanical evaluations are needed for clinical application, the biological capabilities of the titanium surface with the biomimetic physical microenvironment created by a simple chemical treatment offer important information for developing smart biomaterial surfaces for regenerative medicine.

## Experimental Section

### Preparation of Titanium Specimens

Titanium machined discs (JIS-TB340H; φ20 mm and 1 mm thickness) and cylindrical mini-implants (JIS-TR270C; φ20 mm and 1 mm length) were purchased (Nishimura Co., Ltd., Fukui, Japan). Custom-made tooth-shaped titanium implants were prepared by milling an Aadva Ti disk GRADE5 titanium block (GC Corporation, Tokyo, Japan) using a GM-1000 computer-aided design and computer-aided manufacturing (CAD-CAM) system (GC Corporation) according to the scanned data of a mandibular second molar of a 5-week-old male Wistar rat. The titanium specimens were washed with acetone, a series of ethanol, and distilled water (DW) before surface modifications. Next, a microroughened surface clinically used as a typical titanium surface integrating with bone tissue was prepared by immersing the cleaned titanium specimens in 67% (w/w) H_2_SO_4_ solution for 75 s at 120 °C.^26^ In addition, nanoroughened and TRC-mimetic titanium surfaces were prepared by alkali-etching treatment using sodium hydrate, as described previously.^26,27^ Briefly, machined titanium specimens were boiled in a sodium hydrate solution for 24 h, washed them with DW, air-dried them overnight, sintered them in a furnace for 1 h at 600 °C, and, finally, air-cooled them. A 5 M sodium hydrate solution at 60 °C was used for the nanoroughened titanium surface and a 10 M solution at 90 °C for the TRC-mimetic titanium surface.

### Preparation of Human Tooth Samples

In this study, 1 upper and 2 lower impacted wisdom teeth discarded as medical wastes were used as human TRC samples. The TRC surface was exposed by removing the remaining PDLs using sodium hypochlorite treatment, as described previously.^60^ Complete removal of PDLs without damage to the TRC surface was confirmed using optical microscopy of decalcified histological sections of separate sample pieces.

The sampling protocol of human wisdom tooth and individual information management were approved by the Ethical Review Committee of Tohoku University Graduate School of Dentistry, Japan (reception no. 23684).

### SEM Analysis

Titanium or TRC specimens were Au-sputter-coated and then analyzed for surface roughness and elemental analysis using an ERA-600 electron probe 3D surface roughness analyzer (ELIONIX INC., Tokyo, Japan) and an EX-94300S4L1Q EDX system (JEOL Ltd., Tokyo, Japan) at an acceleration voltage of 10 keV. A 3D image was obtained by integrating the calculated inclination angles of the sample surface from the signal intensity ratios of the secondary electrons detected using secondary electron detectors. Roughness parameters were measured under a cutoff value of 6 μm and a measurement length of 12 μm. Vertex extraction images were also obtained by extracting points higher than the surroundings using the triangle division method based on 3D shapes and evaluated the horizontal spatial distribution pattern of dots on the vertex extraction images using a *WinRoof* image analyzer (MITANI Corporation, Tokyo, Japan). Voronoi diagrams for evaluating randomness were prepared using Voronoi tessellation by drawing a perpendicular bisector on a straight line connecting adjacent points and dividing the nearest region of each point.^24^ In addition, the quadrat method was used to evaluate the dot distribution anisotropy by counting the number of dots in each section of the image divided into 18 sections. Randomness and anisotropy were calculated by dividing the standard deviation in the area of polygons in the Voronoi diagrams or in the number of dots within a section by the mean value, respectively. Three areas per sample were measured by a blinded technician.

The hPDLC culture on day 30 on a titanium surface was fixed in 10% neutral buffered formalin for 30 min. The culture samples on titanium disks were washed with DW, air-dried, and then carbon-sputter-coated. Secondary electron observation and elemental analysis were performed on hPDLC culture using an XL30 SEM system (Philips, Eindhoven, The Netherlands) and an EX-94300S4L1Q EDX system at an acceleration voltage of 10 keV. Finally, elemental analysis was performed by randomly selecting 20 × 25 μm^2^ regions.

### Nanoindentation

The microhardness and microelastic modulus of TRC and titanium surfaces were measured using a Nano Indentation Tester ENT-1100b nanoindenter (ELIONIX INC.). A diamond indenter was used to press the sample surface with a maximum load of 0.2 mN at a loading time of 10 s, and the pressing depth was continuously measured using a high-resolution displacement meter. Subsequently, the sample was unloaded at the same time as loading after a 5 s pause. Ten indentation sites were selected under an optical microscope by a blinded technician. Finally, indentation microhardness and elastic modulus were calculated on the basis of established equations by ISO 14577 Part 1; values at measurement points with disturbed load–displacement curves were excluded.

### TEM Analysis

An ultrathin longitudinal section of nanoroughened and TRC-mimetic titanium surfaces was prepared using the ion-milling method for metal specimens.^61^ The testing titanium material was bonded with a dummy lining material using epoxy resin. Next, the test material was shaped using a cutting machine, thinned along the longitudinal sectional direction using mechanical polishing, and ultrathinned on a fixing mesh using a Gatan PIPS691 ion-milling machine (Gatan, Pleasanton, CA, USA).

Next, an ultrathin longitudinal section of an epoxy resin–embedded histological sample at the TRC-mimetic titanium implant–PDL interface in the subrenal capsule transplantation model was prepared using an FIB processing machine equipped with a FEI Versa 3D DualBeam SEM system (Thermo Fisher Scientific Materials and Structural Analysis Division, Hillsboro, OR, USA) and a PP3000T cryo-SEM preparation system (Quorum Technologies Ltd., Lewes, England). The entire surface of the sample was covered with a carbon vapor deposition film, and the processing site was protected using a catalytic chemical vapor deposition film. Next, micropieces including the tissue–titanium implant interface were extracted under SEM observation. After placing the micropieces on a molybdenum-based table for TEM observation, they were ultrathinned to a thickness that would allow the electron beam to pass through while lowering the accelerating voltage from 30 to 5 kV using a gallium ion beam. The ultrathin film processing was performed while cooling the sample stage to −120 °C. Immediately before TEM, the ultrathin longitudinal titanium section was electron-stained with 2% uranium acetate and a mixture of 1% lead acetate, 1% lead nitrate, and 1% lead citrate.

Bright-field observation, selected area electron diffraction, and EDX of the ultrathin longitudinal titanium section were performed using a Hitachi HF2000 TEM system (Hitachi High-Tech Corporation, Tokyo, Japan) equipped with a JEOL JEM-2100F EDX spectroscope (JEOL Ltd.) at an acceleration voltage of 200 keV. The crystallographic characteristics of the nanoroughened and TRC-mimetic titanium surfaces were determined by cross-checking the lattice spacing value obtained from the ED pattern with the powder diffraction file for titanium oxide (International Centre for Diffraction Data, Newtown Square, PA, USA).

### FTIR Spectroscopy

Infrared spectra of titanium surfaces were obtained using an IRT7000 FTIR linear array imaging microscope (JASCO Corporation, Tokyo, Japan). Microreflection spectrum was recorded at 4000–650 cm^–1^ under a 4 cm^–1^ spectral resolution, 500 accumulations, and 50 μm^2^ aperture. Background correction was performed on the basis of the machined surface spectrum.

### Animals

Mandibular bones harvested from 5-week-old male Wistar rats (Tokyo Laboratory Animals Science Co., Ltd., Tokyo, Japan) were used to make decellularized mandibular jawbone with a PDL matrix. Eight-week-old male Wistar rats and 13-week-old male Sprague–Dawley rats (Japan SLC, Inc., Shizuoka, Japan) were used as in vivo models of subrenal capsule transplantation and oral implantation surgery, respectively.

All rat experiments, care, and surgical procedures were approved by the Institutional Laboratory Animal Care and Use Committee of Tohoku University, Japan (protocol no. 2018DnA-048), and the Animal Care Ethics Committee of Tokyo Medical and Dental University, Japan (approval no. 0170020A).

### Subrenal Capsule Transplantation of Decellularized Mandibular Jawbones and PDL Matrices

Decellularized mandibular jawbones with remaining PDL matrices, were prepared as described previously.^23^ Briefly, rat mandibular bones were pressurized for 10 min at 1000 MPa and 10 °C using a Dr. CHEF cold isostatic pressurization machine (Kobe Steel, Kobe, Japan). Pressurization and decompression were then performed at 490 MPa/m. Next, the mandibular bones were incubated for 4 weeks at 37 °C in saline containing 0.2 mg/mL of DNase I (Roche, Indianapolis, IN, USA), 0.5 M MgCl_2_, and antibiotics. Subsequently, the mandibular bones were treated with saline containing 80% ethanol and incubated them for 3 days at 37 °C with shaking, followed by pure saline for 3 days at 37 °C. The decellularized mandibular jawbones were then treated with detergent in order to pull the teeth out, keeping the PDL matrix on the mandibular bones. Briefly, after incubation in 0.3% sodium dodecyl sulfate solution with moderate shaking for 12 h, the teeth were extracted using a pair of forceps. Next, the incisors and the mandibular body were trimmed from the decellularized mandibular jawbones, leaving only the alveolar bone with remaining PDLs in the range of three molars. The decellularized jawbone with decellularized teeth or a tooth-shaped titanium implant with a machined, nanoroughened, or TRC-mimetic titanium surface (Figure S4b) inserted into a tooth extraction socket was embedded in 100 μL of collagen gel (Native Collagen Acidic Solutions I-AC; KOKEN Co., Ltd., Tokyo, Japan) according to the manufacturer’s instructions. The embedded decellularized matrix–tooth/titanium implant complex was incubated for 3 h at 37 °C to aggregate the collagen gel.

Next, a 5–10 mm longitudinal incision was made on the *margo lateralis* of the exposed kidney and detached the renal capsule from the renal cortex to create a pocket for transplantation at the *facies ventralis* side. Subsequently, the decellularized matrix–tooth/titanium implant complex was transplanted into the pocket, with the coronal side toward the renal capsule, and sutured peritoneal membrane and skin with 5–0 absorbable Vicryl and 4–0 silk, respectively. To label mineralizing tissue, we intraperitoneally administered a calcein solution (MP Biomedicals, Inc., Irvine, CA, USA) at a dose of 0.4 mg/100 g body weight weekly after week 4 post-transplantation. Finally, samples were collected at week 8 post-transplantation for histological assessment.

### Oral Surgery for Placements of a Dental Implant and an Orthodontic Device

Occlusal cusps of the rat upper-left first molar were reduced using a dental diamond bar to make free for occlusal contacts. After 1 week, the left first molar was extracted and then a cylindrical titanium mini-implant with a microroughened or TRC-mimetic titanium surface was placed into the mesial extraction socket (Figure S5). Next, the gingival flaps were sutured with 5–0 silk with the implant submerged and intraperitoneally administered a calcein solution weekly after implant placement in order to label mineralizing tissue. Finally, the maxillary bones were collected at weeks 2 and 4 postplacement for μCT and histological assessment of peri-implant tissue.

Biological responses to an externally applied force were also evaluated as a functional analysis of peri-implant tissue using the experimental orthodontic rat model, as described previously.^62^ Briefly, a nickel–titanium-based memory metal coil (YOSHIMI Inc., Aichi, Japan) was connected to the titanium implant at week 2 postplacement (Figure S5). The opposite end of the coil was then tied to the incisors as an anchor to apply an initial contractile force of 20 gf on the implant in a distal-to-mesial direction; an initial contractile force of 40 gf was applied on an intact upper-left molar as a control (the healthy periodontium). The contractile force was applied for 10 days. To measure the mesial displacement of teeth or implants, the rat maxillary dentition was recorded before and after the experimental orthodontic treatment using a dental hydrophilic silicone impression material.^63^ The silicone mold of the rat maxillary dentition was filled with Oken Epok 812 epoxy resin (Okenshoji Co., Ltd., Tokyo, Japan), which was defoamed in a negative pressure chamber (−1 Pa) and then polymerized for 12 h at 60 °C. Next, μCT was used to measure the linear distance on the epoxy model between the mesial edge of the second molar and the notch mark of the titanium implant or the distal edge of the first molar. The mesial displacement of teeth or implants was determined by the difference between the distance before and after the experimental orthodontic treatment. After μCT scanning, histological assessment of the periodontal and peri-implant tissues was performed.

### μCT Analysis

The maxilla was fixed in 10% neutral buffered formalin (FUJIFILM Wako Pure Chemical Corporation, Osaka, Japan) for 3 days at 4 °C. The specimen was analyzed using a ScanXmate-E090 device for 3D μCT imaging (Comscan Tecno Co., Ltd., Kanagawa, Japan) and TRI/3D-BON bone structure analysis software (Ratoc System Engineering, Tokyo, Japan). The maxilla specimens were X-rayed at an energy level of 80 kVp and a current of 60 μA through a 1 mm-thick brass filter. More than 300 CT cross-sectional images were obtained in the longitudinal sectional direction of the implants or teeth at an isotropic voxel size of 20 μm. To analyze the mesial displacement of implants caused by an externally applied force, the epoxy models before and after orthodontic force application were X-rayed at an energy level of 60 kVp and a current of 90 μA at an isotropic voxel size of 30 μm without a filter. The 3D images obtained were reconstructed using the calibration curves for bone mineral content obtained by scanning a HAp phantom under the same X-ray conditions. The area of interest for analysis was set within 400 μm of the tooth root or the implant surface, from 400 μm outside the apex of the tooth root or the implant to the alveolar crest. Specific thresholds for bone tissue, teeth, and implants were determined by superimposing segmented images over the original gray scale X-ray images. The expected PDL space was set in the region within 200 μm from the implant surface. The tendency of the PDL response around the implants toward regeneration of the periodontium or osseointegration was also assessed by determining the volume of voids around the implants and the occupancy of mineralized structure within the expected PDL space. In addition, the mesial displacement of implants after orthodontic force application was analyzed by measuring the centers of the mesial marginal ridge on the second molar and the notch on the implant on 3D reconstruction images. A typical 3D cross-sectional image of a longitudinal section at the center of the teeth or implants was obtained.

### Processing of Nondecalcified Sections and Histomorphometry

Sample tissues taken from the subrenal capsule transplantation or the oral implantation model were immersed in 70% ethanol after fixation in 10% neutral buffered formalin. Next, the samples underwent en bloc staining with Villanueva osteochrome bone stain solution (Polysciences, Inc., Warrington, PA, USA) for 2 weeks while degassing at −1 Pa for 30 min daily. Villanueva osteochrome bone stain was used as a counterstain for the calcein vital stain for mineralizing tissue^64^ because it provides a dark-blue or purple color to cells and soft tissue but translucency on mineralized tissue. After washing and then dehydrating with a series of ethanol, acetone, and xylene, the samples were embedded in poly(methyl methacrylate) (FUJIFILM Wako Pure Chemical Corporation) or epoxy resin for histological and histomorphometrical evaluation or TEM analysis, respectively. The samples were sectioned at the center of the teeth or implants in the longitudinal or cross-sectional direction using an SP1600 saw microtome machine (Leica Camera AG, Wetzlar, Germany). Next, each section was ground to a thickness of 50 μm. The nondecalcified resin-embedded sections were observed under a BZ-9000 all-in-one fluorescence microscope (KEYENCE CORPORATION., Tokyo, Japan). The calcein signals were superimposed on the bright-field image. Next, the capability of the decellularized tooth or implant surface to form a TRC was assessed by quantifying the percentage of the calcein-positive area on the tooth or implant surface. The sections in the subrenal capsule transplantation model were further stained with Villanueva Goldner stain to color mineralized (green) or nonmineralized (red-purple) tissue in different colors after optical and fluorescent microscopy. Finally, the capability of the decellularized tooth or implant surface to support induction of dentoalveolar fibrous joints was assessed by quantifying the fiber area percentage in the PDL space.

### Processing for Decalcified Sections and Histological Staining

A part of the samples in the oral implantation model was processed for decalcified sections. After fixation in 10% neutral buffered formalin, the tissue samples were delipidated with a series of ethanol and acetone and then decalcified them using 20% ethylenediaminetetraacetic acid (EDTA) solution (FUJIFILM Wako Pure Chemical Corporation) for 14 days. Next, the cylindrical titanium mini-implant was removed after decalcification and the paraffin-embedded specimen was sectioned at the center of the first molar tooth or implant to a thickness of 5 μm in the longitudinal direction.

Immunohistochemical staining for periostin and osteocalcin was performed to detect PDL and active osteoblasts markers, respectively, in the peri-implant tissue. After deparaffinization, the sections were boiled for 30 min in 20 mM Tris-HCl with 0.05% Tween 20 in a 95 °C boiling chamber for antigen activation. After blocking endogenous peroxidase activity and nonspecific reaction using 3% hydrogen peroxide and skim milk, the sections were incubated for 9 h at 4 °C in 1/200 or 50 diluted rabbit polyclonal antiperiostin (ab14041; Abcam plc., Cambridge, England) or antiosteocalcin (sc-30045; Santa Cruz Biotechnology, Inc., Dallas, TX, USA) antibody, respectively. Subsequently, the sections were reacted with the peroxidase-conjugated antirabbit second antibody (MK205; Takara Bio Inc., Shiga, Japan) for 60 min and then colored them using 3,3′-diaminobenzidine solution (MK210; Takara Bio Inc.) for 10 min at room temperature (RT).

TRAP staining was performed using the TRAP/ALP Stain Kit (FUJIFILM Wako Pure Chemical Corporation) according to the manufacturer’s instructions. Finally, the sections were incubated in a mixed solution of tartaric acid and acid phosphatase substrate for 30 h at 37 °C, with methyl green used as a counterstain for the nucleus.

### hPDLC Culture

Normal human PDL-derived fibroblastic cells (CC-7049; Lonza Walkersville, Inc., Basel, Switzerland) were purchased and expanded them in Minimum Essential Medium, Alpha Modification (α-MEM; Gluta MAX, no nucleosides; Thermo Fisher Scientific) supplemented with 10% fetal bovine serum (FBS; Japan Bioserum, Hiroshima, Japan), 100 U of penicillin, and 100 μg/mL of streptomycin (FUJIFILM Wako Pure Chemical Corporation) at 37 °C in a 5% CO_2_ atmosphere. The hPDLCs after 5–7 passages were used. After 80% confluence, the cells were detached with 0.25% trypsin/1 mM EDTA and seeded them onto titanium disks on 12-well culture-graded polystyrene plates at a density of 3.0 × 10^4^ cells/cm^2^ in cementogenic differentiation medium consisting of α-MEM supplemented with 10% FBS (GIBCO FBS 10082147; Thermo Fisher Scientific), 1 × 10^–8^ M dexamethasone, 10 mM Na-β-glycerophosphate, 50 μg/mL of ascorbic acid, 100 U of penicillin, and 100 μg/mL of streptomycin at 37 °C in a 5% CO_2_ atmosphere.

### Calcium Deposition Assay

The hPDLC cultures on polystyrene and titanium substrates were washed at days 10 and 30 with phosphate-buffered saline (PBS) and incubated them overnight in 1 mL of 0.5 M HCl solution with gentle shaking. Next, the solution was mixed with *o*-cresolphthalein complexone in alkaline medium (Calcium Assay Kit; Cayman Chemical Company, Ann Arbor, MI, USA) to produce a purple cresolphthalein complexone complex. The color intensity was measured in terms of absorbance at a wavelength of 570 nm using an enzyme-linked immunosorbent assay (ELISA) reader. In addition, to calculate the calcium concentration of the samples, the corrected absorbance value of each sample was substituted into the equation obtained from the linear regression of the standard curve of the standard calcium solution, and the calcium concentration was converted to the calcium density per unit culture area.

### TNAP Activity Assay

The TNAP activity of the hPDLC cultures was analyzed on polystyrene and titanium substrates at days 14 and 24 using colorimetry-based assay (LabAssay ALP; FUJIFILM Wako Pure Chemical Corporation). Briefly, the cells were rinsed with PBS and incubated in *p*-nitrophenylphosphate solution for 15 min at 37 °C. The absorbance of *p*-nitrophenol released as a result of the enzymatic reaction was measured at a wavelength of 405 nm using an ELISA reader. Finally, TNAP activity was evaluated on the basis of the defined unit as the release of 1 nmol of *p*-nitrophenol/min at pH 9.8 and 37 °C.

### Quantification of ePi and ePPi

The hPDLC cultures were incubated on polystyrene and titanium substrates at days 20 and 30 in DW for 24 h at 37 °C. The amounts of ePi and ePPi in the supernatants were measured on the basis of the malachite green–molybdate-binding reaction in the presence of Pi and the enzymic reaction for adenosine monophosphate (AMP) phosphorylation to adenosine triphosphate (ATP) using PPi, respectively. For ePi detection, the diluted supernatant was mixed with malachite green and molybdenum acid solutions (BioAssay Systems, Hayward, CA, USA) and incubated the mixture in a shaker for 30 min at RT. Absorbance was measured at a wavelength of 620 nm for the dark-blue color of the Pi–malachite green–molybdate acid complex using an ELISA reader. For ePPi detection, the supernatant was mixed with converting reagents containing phosphotransferases and pyrophosphatases (PPiLight inorganic pyrophosphate assay; Lonza Walkersville, Inc.) and incubated the mixture for 30 min at RT to convert AMP to ATP. Next, a detection reagent containing luciferase was added to produce luminescence from the newly formed ATP and luciferin and the mixture was further incubated for 30 min. The luminescence intensity was measured using a luminometer on 0.1 s integration/sample. Finally, the ePi and ePPi concentrations were calculated by substituting the corrected absorbance or luminescence value for each sample into the equation obtained from the linear regression of the standard curve of standard Pi and PPi solutions.

### Reverse Transcription Polymerase Chain Reaction (RT-PCR) Analysis

The hPDLC cultures on polystyrene and titanium substrates were treated with TRIzol (Ambion/Life Technologies, Carlsbad, CA, USA) at days 1, 5, 10, 20, and 30. Total RNA isolation and purification were performed using a spin column (RNeasy Mini Kit; Qiagen, Germany). Next, complementary DNA (cDNA) with 1 μg of total RNA was synthesized using PrimeScript II reverse transcriptase in the presence of the oligo (dT) primer (PrimeScript II first strand cDNA Synthesis Kit; Takara Bio Inc.) after removal treatment for DNase and cation (DNA-free; Thermo Fisher Scientific). PCR analysis was performed using the StepOnePlus real-time PCR system (Applied Biosystems, Foster City, CA, USA) and the Thunderbird SYBR qPCR Mix (Toyobo, Osaka, Japan). Finally, the data were quantitatively analyzed using the comparative cycle time (ΔΔCT) method. Glyceraldehyde 3-phosphate dehydrogenase (GAPDH) was used as a housekeeping gene. Table S1 lists the primer sequences used in this study.

### Statistical Analysis

The mean values of each group for all surface properties for the horizontal pattern of vertex distribution, vertical roughness, and micromechanical properties in multiple dimensions were plotted. Next, the analogousness of surface properties between TRC and titanium surfaces by measuring the Euclidean distance between each point in the multidimensional plots was evaluated using the KNN method (*k* = 6). Power analysis for all quantitative data was calculated in in vivo rat experiments with a type-one error rate of 0.05 and 80% power to claim noninferiority of TRC-mimetic titanium implants to decellularized teeth or osseointegrated titanium implants. One-way analysis of variance (ANOVA) was used to assess differences among multiple experimental groups, while two-way ANOVA was used to assess the interaction between the differences in the types of titanium surfaces and the culturing or healing period. When appropriate, post hoc Dunnett’s test or Tukey’s honestly significant difference (HSD) test was used. In addition, Spearman’s correlation test was used to evaluate the correlation between various factors observed on the titanium surface in hPDLC culture experiments. In SEM analysis and nanoindentation, three independent tooth samples were subjected to a series of measurements, whereas one sample was used on each titanium surface where uniform quality was previously confirmed.^26,27^ All in vitro experiments were performed in at least three independent cell batches. *p* < 0.05 was considered statistically significant. All statistical analyses were performed using *IBM SPSS Statistics 21* (IBM Japan, Ltd., Tokyo, Japan) and G*Power 3.1.9.7 (Heinrich-Heine-Universität Düsseldorf, Düsseldorf, Germany).

## Supporting Information Available

Topographic and micromechanical features of TRC and titanium surfaces, comparison of topographical and micromechanical properties of TRC among individuals, crystallographic and chemical features of nanoroughened titanium surfaces, materials and their features in the subrenal capsule transplantation model, and experimental procedures in oral implant and orthodontic surgeries (PDF)
