# Supplementary material for: Titanium Nanosurface with a Biomimetic Physical Microenvironment to Induce Endogenous Regeneration of the Periodontium
Source: ACS Appl Mater Interfaces. 2022 Jun 13;14(24):27703–19. doi: 10.1021/acsami.2c06679 (PMC9231364; doi:10.1021/acsami.2c06679)
Supplement: Supplementary file 1 — am2c06679_si_001.pdf [file am2c06679_si_001.pdf]

Supporting information

The Titanium Nanosurface with Biomimetic Physical Microenvironment to Induce Endogenous Regeneration of the Periodontium

Masahiro Yamada[1], Tsuyoshi Kimura[2], Naoko Nakamura[3], Jun Watanabe[1], Nadia Kartikasari[1], Xindie He[1], Watcharaphol Tiskratok[1], Hayato Yoshioka[4], Hidenori Shinno[4] and Hiroshi Egusa[1, 5]*.*

[1] Division of Molecular and Regenerative Prosthodontics, Tohoku University Graduate School of Dentistry, Sendai, Miyagi, 980-8575, Japan
[2] Institute of Biomaterials and Bioengineering, Tokyo Medical and Dental University, Chiyoda-ku, Tokyo, 101-0062, Japan
[3] Department of Bioscience and Engineering, College of Systems Engineering and Science, Shibaura Institute of Technology, Saitama, Saitama, 135-8548, Japan
[4] Laboratory for Future Interdisciplinary Research of Science and Technology, Tokyo Institute of Technology, Yokohama, Kanagawa, 152-8550, Japan
[5] Center for Advanced Stem Cell and Regenerative Research, Tohoku University Graduate School of Dentistry, Sendai, Miyagi, 980-8575, Japan

*Address corresponding to:
Masahiro Yamada, DDS, PhD
Division of Molecular and Regenerative Prosthodontics, Tohoku University Graduate School of Dentistry, 4-1, Seiryouchou, Aoba-ku, Sendai, 980-8575, Japan
tel: (+81) 022-717-8363; fax: (+81) 022-717-8367
E-mail: masahiro.yamada.a2@tohoku.ac.jp

Hiroshi Egusa, DDS, PhD
Division of Molecular and Regenerative Prosthodontics, Tohoku University Graduate School of Dentistry, 4-1, Seiryouchou, Aoba-ku, Sendai, 980-8575, Japan
tel: (+81) 022-717-8363; fax: (+81) 022-717-8367
E-mail: egu@tohoku.ac.jp

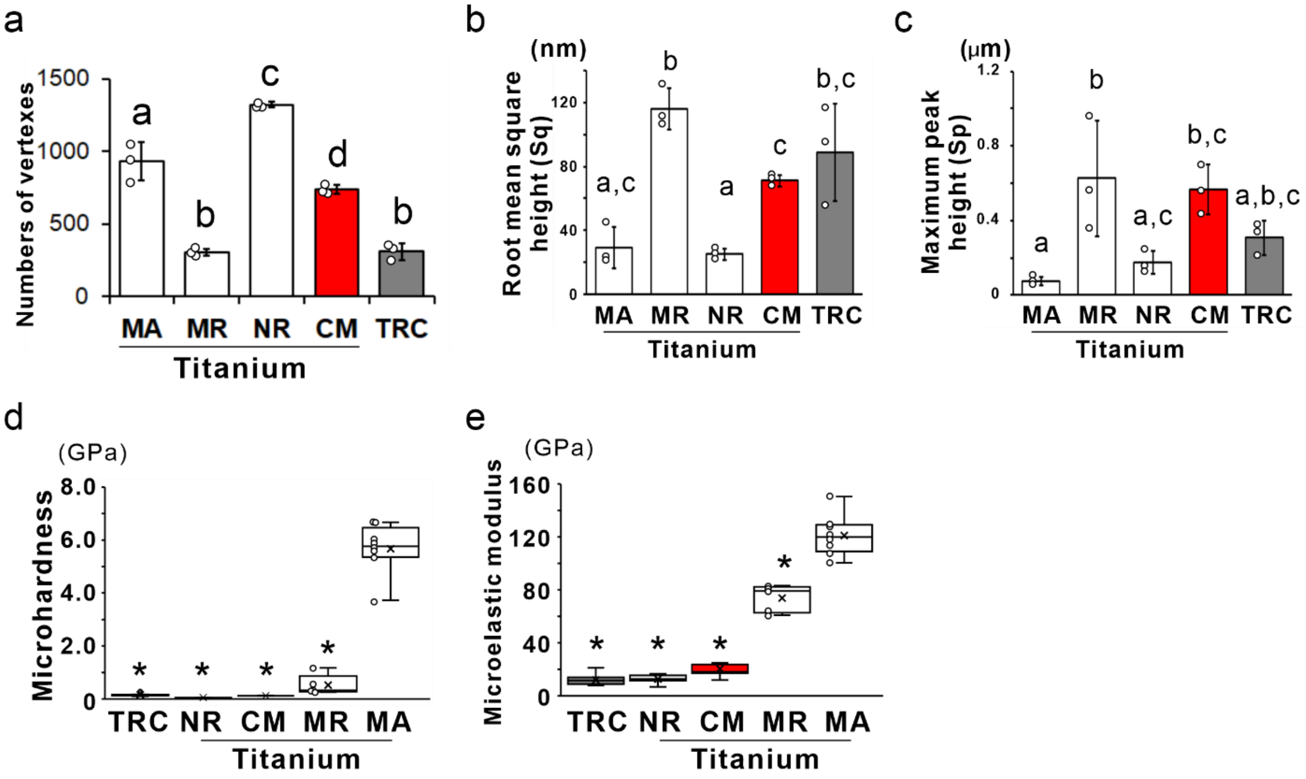

Figure S1. Topographic and micromechanical features of TRC and titanium surfaces. Number of vertexes (**a**) and vertical roughness parameteres such as root mean square height (**b**) and maximum peak height (**c**) on machined (MA), micro-roughened (MR), nanoroughened (NR), and TRC-mimetic (CM) titanium and TRC surfaces were measured by analyzing vertex extraction SEM images, Voronoi diagrams and 3D SEM images. Data presented as the mean ± SD with dot plots. Different letters indicate statistically significant differences between them ($P < 0.05$; Tukey's HSD test). Microhardness (**d**) and microelastic modulus (**e**) measured by nanoindentation on MA, MR, NR, and CM titanium and TRC surfaces. Five to ten independent spots on one sample. Data presented as box and dot plots with a mean point. *Statistically significant differences between the MA titanium surface versus other implant surfaces (**b** and **c**) ($P < 0.05$; Dunnett's test). TRC, tooth root cementum; SEM, scanning electron microscopy; HSD, honestly significant difference; SD, standard deviation.

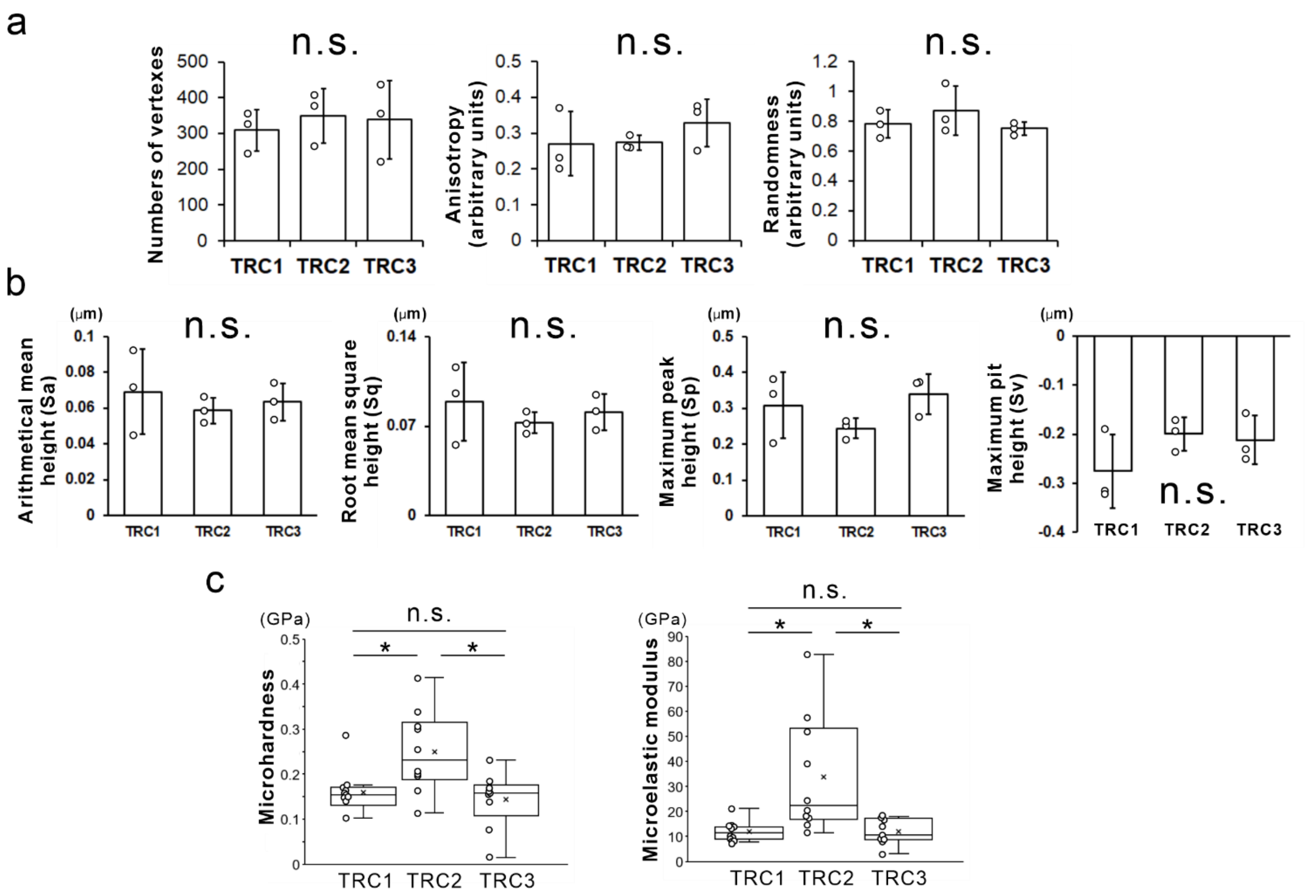

Figure S2. Comparison of topographical and micromechanical properties of TRC among individuals

(**a**) Number of vertexes and anisotropy and randomness in the vertex distribution on the TRC measured by analyzing vertex extraction SEM images and Voronoi diagrams. (**b**) Vertical roughness parameters (lower histograms) on three TRC surfaces under 3D SEM. Three independent regions on one sample. Data presented as the mean ± SD with dot plots. n.s. indicates no statistically significant differences between them ($P > 0.05$; one-way ANOVA). (**c**) Microhardness and microelastic modulus measured by nanoindentation on three TRC surfaces. Nine to ten independent spots on one sample. Data presented as box and dot plots with a mean point. *Statistically significant differences between TRC2 and others ($P < 0.05$; Tukey's HSD test). n.s. indicates no statistically significant differences between TRC1 and TRC3 ($P > 0.05$; Tukey's HSD test). TRC, tooth root cementum; SEM, scanning electron microscopy; SD, standard deviation; ANOVA, analysis of variance; HSD, honestly significant difference.

Nanoroughened titanium

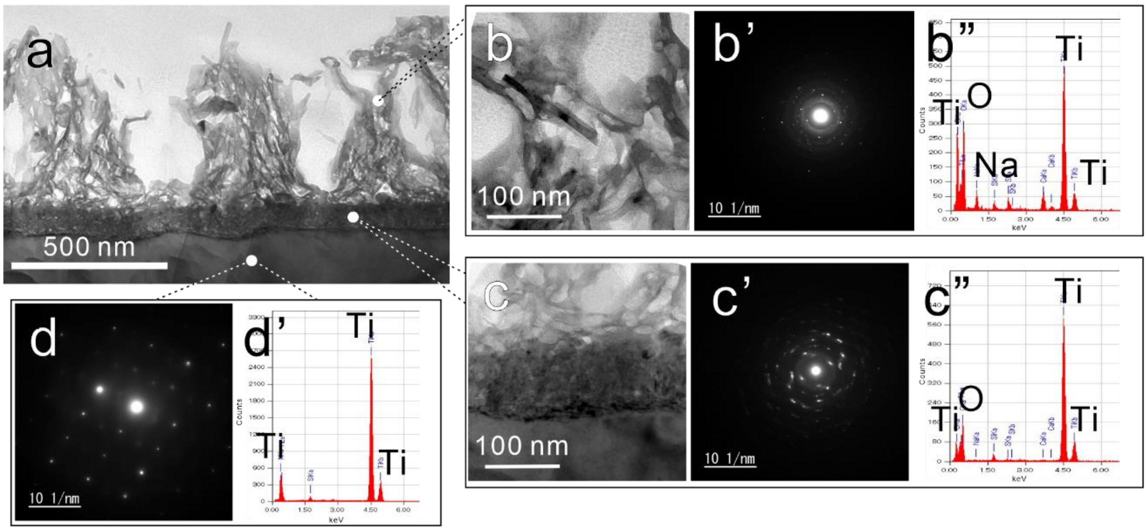

Figure S3. Crystallographic and chemical features of nanoroughened titanium surfaces
Bright-field (**a**–**c**) and SAED (**b'**, **c'**, and **d**) images and EDX profile (**b"**, **c"**, and **d'**) on the ultrathin titanium longitudinal section of nanoroughened titanium surfaces under TEM. Data were taken from each spot showing the corresponding typical features on superficial (**b**–**b"**) and transitional (**c**–**c"**) layers and titanium base (**d** and **d'**). SAED, selected area electron diffraction; EDX, energy-dispersive X-ray spectroscopy; TEM, transmission electron microscopy

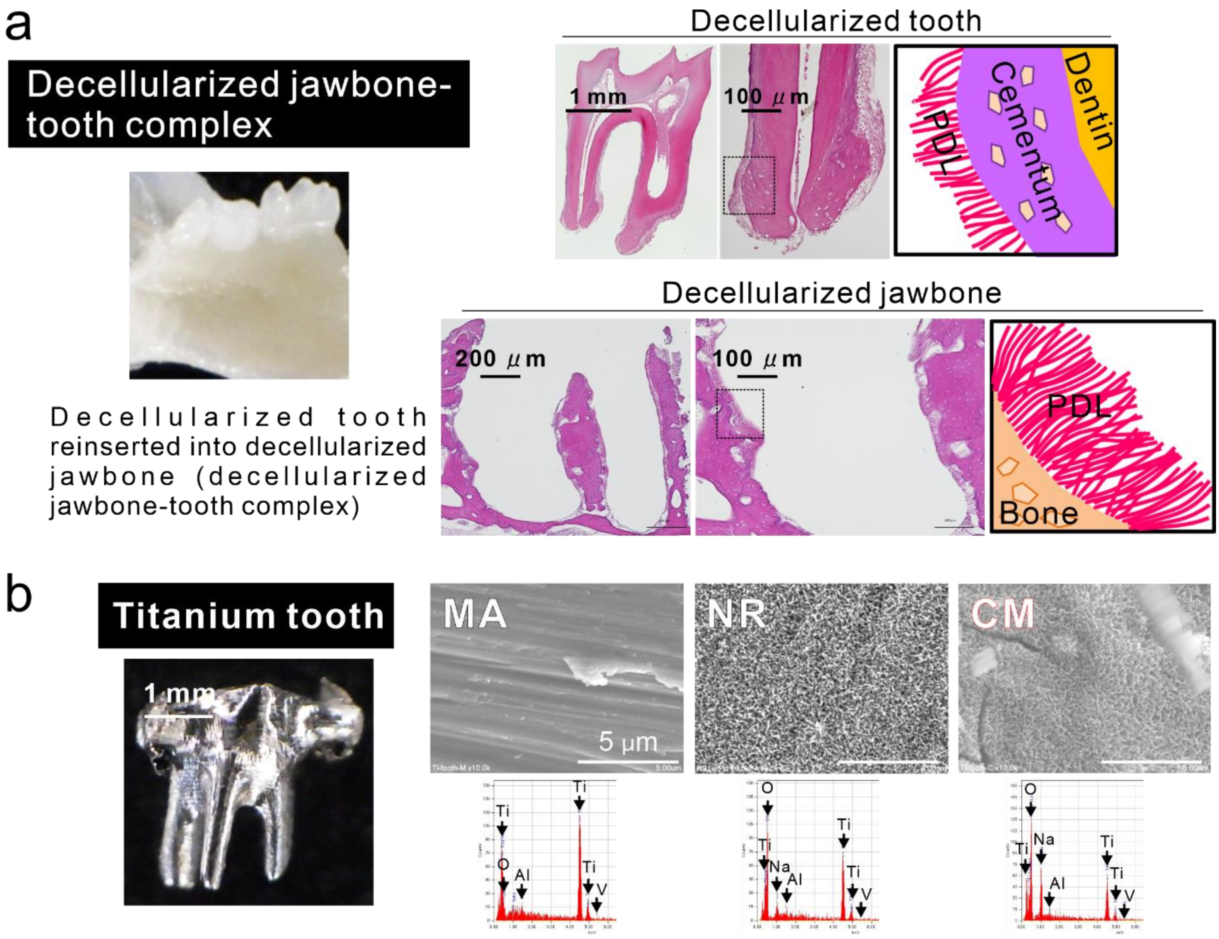

Figure S4. Materials and tissue-titanium interface elemental features in the subrenal capsule transplantation model

The translucent decellularized tooth and jawbone obtained from 5-week-old male Wistar rats do not have any cells but maintain dense residual PDL fibers (**a**, macroscopic picture, optical microscopic images of H&E-stained decalcified sections and schemes). Black dashed squares in H&E-stained section images indicate the location of the corresponding scheme. (**b**) Appearance, secondary electron SEM images and EDX profiles on titanium teeth having machined (MA), nanoroughened (NR), and TRC-mimetic (CM) surfaces. Note (**b**) that titanium implants with the same shape as the rat molar have typical topographycal features as seen on SEM images of pure titanium samples in **Figure 1**. PDL, periodontal ligament; H&E, hematoxylin-eosin; TRC, tooth root cementum; SEM, scanning electron microscopy; EDX, energy-dispersive X-ray spectroscopy.

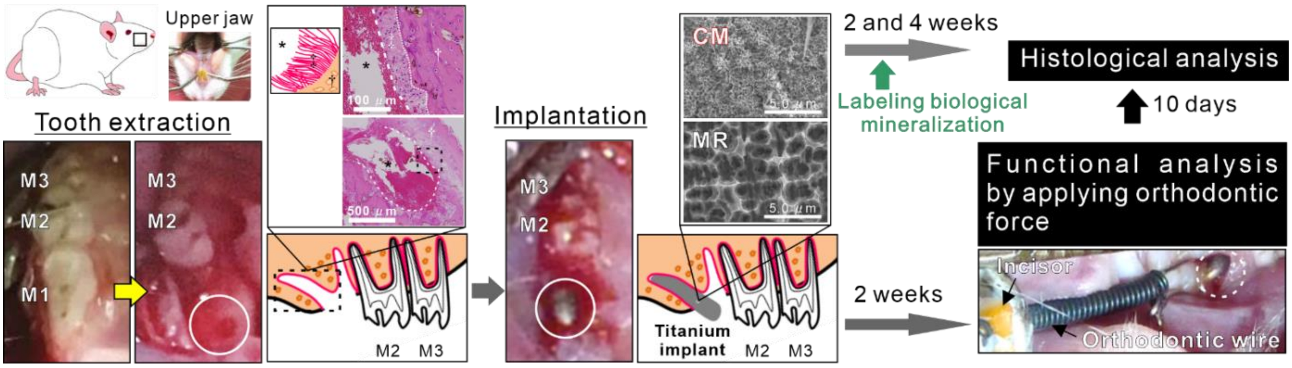

Figure S5. Experimental procedures in oral implant and orthodontic surgeries

The upper-left first molar (M1) of 13-week-old male Sprague–Dawley rats was extracted after tooth reduction. A titanium cylindrical mini-implant with a microroughened (MR) or TRC-mimetic (CM) surface was placed into the mesial extraction socket where the PDL remains on the alveolar bone (macroscopic pictures of the rat oral cavity, optical microscopic images of H&E-stained decalcified sections, SEM images for each implant surface and scheme). After 2 and 4 weeks healing together with intraperitoneal administration of a calcein solution weekly after implant placement to label mineralizing tissue, the peri-implant tissues were histologically evaluated. Alternatively, after 2 weeks of healing, a nickel-titanium-based memory metal coil was connected to the titanium implant by tying the opposite end of the coil to the incisors as an anchor. An initial contractile force of 20 or 40 gf in a distal-to-mesial direction was applied on the titanium implant or an intact upper-left molar, respectively. After 10 days of contractile loading, peri-implant and periodontal tissues were histologically evaluated. *Extraction socket cavity; †alveolar bone; ‡PDL. White circles in macroscopic pictures indicate the implant placement location. White dotted and dashed lines in H&E-stained section images indicate borders between the alveolar bone and the PDL and between the PDL and the extraction socket cavity, respectively. Black dashed enclosures indicate the corresponding regions for H&E-stained section images. PDL, periodontal ligament; TRC, tooth root cementum; SEM, scanning electron microscopy; H&E, hematoxylin-eosin.

Table S1 Primer list

Encoded protein name (gene name)	Primers (Fw, forward; Rv, reverse)	Product size (bp)	Accession number
Cementum protein 1 (*CEMP1*)	Fw: 5'-ACCCCTTAGGAAGTGGCTG-3'<br>Rv: 5'-TGAGAACCTCACCTGCCTC-3'	100	NM_001048212.3
Bone sialoprotein (*BSP*)	Fw: 5’-GCGAAGCAGAAGTGGATGAAA-3’<br>Rv: 5’-TGCCTCTGTGCTGTTGGTACTG-3’	65	NM_004967.4
Sclerostin (*SOST*)	Fw: 5'-CGGGCGGAGAACGGA-3'<br>Rv: 5'-CGGCCCATCGGTCACGTA-3'	108	NM_025237.3
Tissue-nonspecific alkaline phosphatase (*TNAP*)	Fw: 5’-CCATCCTGTATGGCAATGG-3’<br>Rv: 5’-CATGGAGACATTCTCTCGTTCA-3’	65	NM_001127501.4
Progressive ankylosis protein (*ANK*)	Fw: 5’-CCTGAAGGTCGCATTAGAGC-3’<br>Rv: 5’-TTACCCTACAGCAACCTGGC-3’	119	NM_054027.6
Ectonucleotide pyrophosphatase / phosphodiesterase 1 (*ENPP1*)	Fw: 5’-TTACCAGGAGACGTGCATAGA-3’<br>Rv: 5’-GGTCAACCTTTTCTCACCACAC-3’	76	NM_006208.3
Glyceraldehyde 3-phosphate dehydrogenase (*GAPDH*)	Fw: 5'- AATCCCATCACCATCTTCCA-3'<br>Rv: 5'- TGGACTCCACGACGTACTCA-3'	82	NM_001357943.2
